# Enhancing groundwater potential mapping in arid regions through integrated microwave remote sensing, statistical analysis, and geophysical-based modeling

**DOI:** 10.1038/s41598-026-64619-0

**Published:** 2026-08-16

**Authors:** Youssef M. Youssef, Khaled S. Gemail, Nada Ashraf Abdel Aziz, Mahmoud M. Senosy, Gad El-Qady, Hafsa M. Atia

**Affiliations:** 1 Geological and Geophysical Engineering Department, Faculty of Petroleum and Mining Engineering, Suez University, Suez, 43518 Egypt; 2 Zagazig University, Environmental Geophysics Lab (ZEGL), Department of Geology, Faculty of Science, Zagazig, 44519 Egypt; 3 Geology Department, Faculty of Science, Assiut University, Assiut, Egypt; 4 National Research Institute of Astronomy and Geophysics (NRIAG), Helwan, Cairo, Egypt; 5 Geology Department, Faculty of Science, Mansoura University, Mansoura, 35516 Egypt

**Keywords:** Groundwater potential modeling, Arid regions, Microwave remote sensing, DC resistivity, Magnetic exploration, Sustainable water management, Climate sciences, Environmental sciences, Hydrology, Solid Earth sciences

## Abstract

The increasing water scarcity in arid regions underscores the urgent necessity to map and manage structurally controlled aquifers (SCAq), which play a pivotal yet underappreciated role as water sources. This study presents an integrative framework that synergistically combines statistical remote sensing methods with geophysical investigations to precisely delineate groundwater potential (GwP) within the Wadi Hodein basin in the Eastern Desert of Egypt. Microwave remote sensing data from Sentinel-1 SAR, ALOS PALSAR, and IMERG were integrated with geophysical techniques, including aeromagnetic surveys and DC resistivity measurements, to enhance predictive accuracy for structurally controlled aquifer characterization. The workflow involved developing ensemble bivariate models, namely EBF-IOE and WOE-IOE, leveraging morphometric, climatic, and hydrogeological datasets, followed by geophysical validations to identify and characterize high-potential zones. The EBF-IOE model exhibited exceptional predictive performance (AUC = 80.11%), highlighting areas predominantly influenced by lineament density, lithologic variations, and fault networks. Geophysical analyses revealed that these promising zones are compartmentalized by NNW-SSE and E-W fault systems, providing insights into local variability in well yield and water quality that statistical models alone could not capture. Aeromagnetic data established a structural context, while DC resistivity measurements quantified aquifer geometry, linking resistivity values (17–111 Ω·m) with productive areas and water salinity levels (824–2120 ppm). This study demonstrates that integrating statistical modeling with geophysical methods is crucial for transitioning from broad regional predictions to a nuanced understanding of SCAqs. The proposed framework presents a scalable model that mitigates risks in groundwater exploration across arid regions globally, thereby promoting sustainable extraction and managed aquifer recharge strategies.

**Supplementary Information:**

The online version contains supplementary material available at 10.1038/s41598-026-64619-0.

## Introduction

Global freshwater security is increasingly threatened by rising water demand, driven by population growth and agricultural expansion, particularly in water-stressed, arid developing nations. Although the annual global renewable freshwater supply is estimated at 37,000 km^3^^1–3^, consumption is projected to surge by 20–30% by 2050^4–6^. This pressure is compounded by the impacts of climate change, which further disrupt the availability and distribution of water resources in these vulnerable regions.

In arid environments, groundwater within structurally controlled aquifers (SCAq) of hard-rock terrains is often the only reliable freshwater source^7,8^. These complex systems, defined by fractured bedrock and weathered overburden, exhibit high spatial heterogeneity^9–12^. When combined with unsustainable abstraction for agriculture, this heterogeneity accelerates aquifer depletion and threatens long-term water and food security^13–17^. Consequently, developing effective strategies for groundwater management in these regions is a pressing global priority.

Accurate assessment of groundwater potential (GwP) in heterogeneous, data-scarce environments requires a comprehensive, integrated approach. Traditional methods, however, encounter significant challenges. Conventional geophysical techniques, such as vertical electrical sounding (VES), provide valuable point-based data but are inherently one-dimensional^18–21^. This limitation makes them difficult and costly to scale for regional characterization, particularly in areas lacking comprehensive borehole data. While aeromagnetic surveys can help map regional structures, their resolution is inadequate for identifying shallow, high-resolution controls on groundwater occurrence, which are essential for understanding local dynamics.

Recent advances in microwave remote sensing, such as Sentinel-1 SAR and ALOS PALSAR, present transformative opportunities by enabling high-resolution, all-weather mapping of critical surface and subsurface features, including lineaments and paleochannels^22–25^. Despite these advancements, a significant research gap persists: there is currently no systematic framework for integrating these powerful remote sensing datasets with traditional hydrogeological and geophysical data. This integration is vital for developing a robust and scalable methodology for GwP assessment^26–28^

Moreover, the impact of local subsurface geological structures on the dynamic groundwater recharge system warrants closer examination. Existing remote sensing data often fail to adequately capture this heterogeneity, particularly in structurally controlled aquifer systems where variations in geology can significantly influence groundwater flow and recharge characteristics. Addressing this gap in understanding is crucial for enhancing the accuracy and reliability of GwP assessments, particularly in complex terrains where both geophysical survey limitations and data scarcity constrain effective resource management.

The challenge of integrating diverse geospatial data for GwP mapping is well-suited to GIS-based multi-criteria decision analysis (MCDA). While techniques like the Analytical Hierarchy Process (AHP) are common, their reliance on subjective expert judgment can introduce bias^29,30^. To overcome this, bivariate statistical models, including Evidential Belief Function (EBF), and Weights of Evidence (WOE), have been effectively employed to quantitatively assess groundwater controls in data-scarce regions^31–37^. The Index of Entropy (IOE) further enhances these approaches by objectively accounting for class-specific dependencies within each predictive factor^38,39^. Recent research demonstrates that ensemble modeling, which combines the strengths of multiple models, can significantly improve prediction accuracy and provide a more robust understanding of GwP^40–42^. Despite these advances, no study has fully leveraged an ensemble of bivariate models to integrate key parameters from Sentinel-1A, specifically VH polarization backscattering and VV-derived lineaments, with other geospatial datasets to explicitly investigate the role of subtle features such as buried channels and lineament-stream intersections in SCAq systems.

This study addresses a fundamental gap by developing and validating an integrative, cost-effective methodology for groundwater potential (GwP) assessment in structurally controlled aquifer (SCAq) systems. This approach integrates open-source remote sensing and climate datasets (Sentinel-1A, ALOS DEM, and IMERG) with relevant ancillary geospatial layers. It then employs an ensemble bivariate modeling framework that combines weights of evidence (WOE) and evidential belief function (EBF) techniques to robustly quantify and fuse predictive signals.

The specific objectives of this research are to: (1) identify and quantify the influence of topographic, lithological, hydrological, and climatic factors on groundwater occurrence; (2) implement and compare ensemble bivariate models to delineate high-potential groundwater zones; and (3) rigorously validate the resulting GwP maps through targeted aeromagnetic and vertical electrical sounding (VES) geophysical surveys. By providing a transferable framework for sustainable groundwater management, this research supports the United Nations Sustainable Development Goals (SDGs), particularly SDG 6 (Clean Water and Sanitation), in water-stressed environments globally.

## Study area characterization

**Fig. 1:**
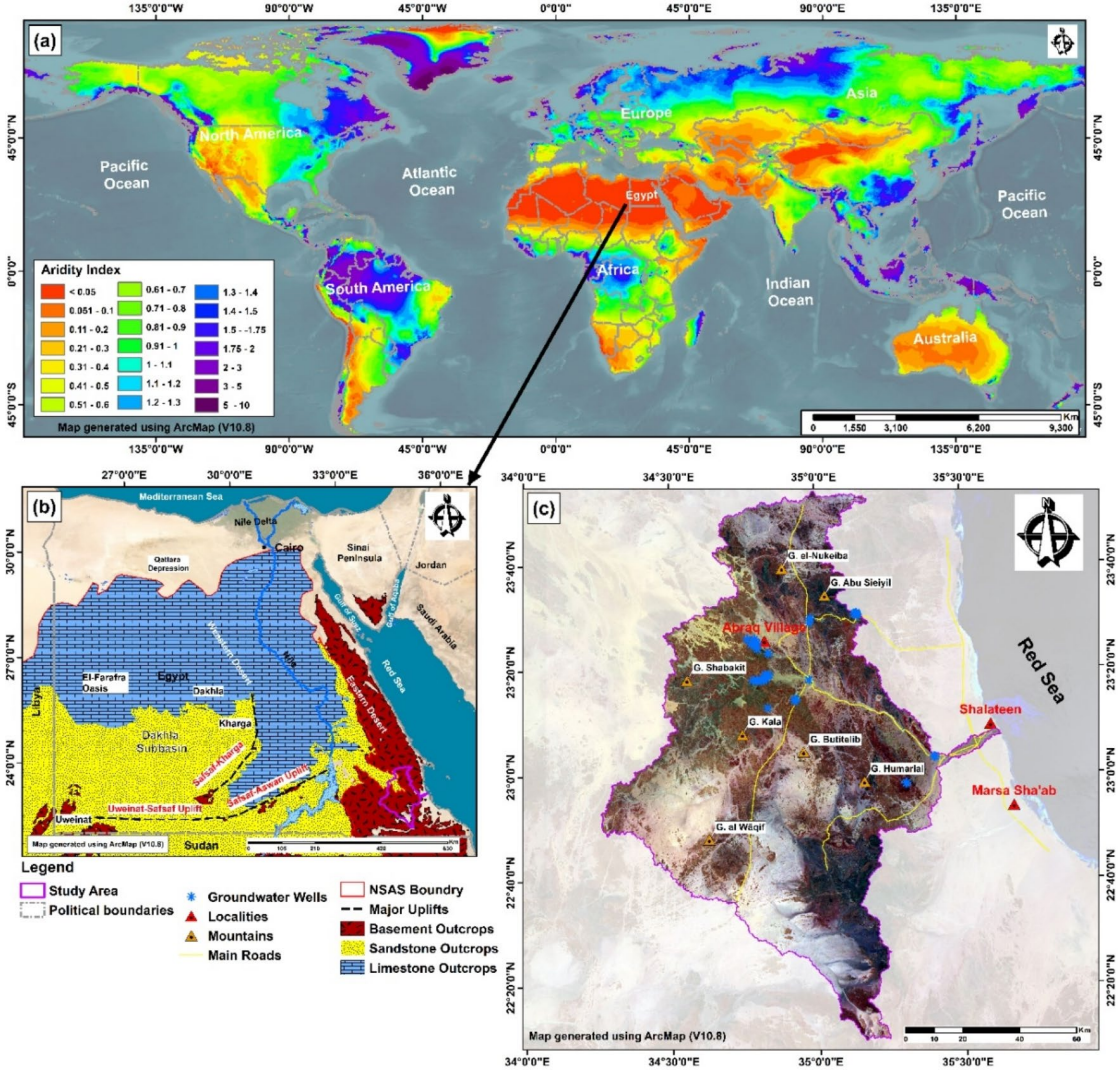
Study area location: (**a**) Global aridity map adapted from the Global Aridity Index and Potential Evapotranspiration (ET_0_) Database v3.1^43^, which is publicly available at 10.6084/m9.figshare.7504448. Cartographic processing and figure preparation were performed using ArcGIS Pro v3.6. (**b**) Location of the study area in southeastern Egypt (purple polygon), showing the distribution of Nubian Sandstone and crystalline basement outcrops. The map was produced using ArcGIS Pro v3.6 (https://www.esri.com/en-us/arcgis/products/arcgis-pro/overview). (**c**) Sentinel-2 true-colour (RGB) image of Wadi Hodein, Shalatein. Sentinel-2 imagery was obtained from the Copernicus Data Space Ecosystem (https://dataspace.copernicus.eu/data-collections/copernicus-sentinel-missions/sentinel-2), and the final map layout was prepared in ArcGIS Pro v3.6 (https://www.esri.com/en-us/arcgis/products/arcgis-pro/overview).

This case study focuses on the Shalatein region, located in Egypt’s South Eastern Desert (SED). This rapidly urbanizing coastal zone highlights the challenges encountered in hyper-arid regions, as depicted in Fig. 1a. The area faces significant water scarcity, driven by complex geological conditions, an arid climate, and seawater intrusion, compounded by a shortage of comprehensive hydrogeological studies^44–49^. Shalatein experiences an arid climate characterized by hot, humid summers, with average temperatures of 37.5 °C, and relatively mild winters.

**Fig. 2:**
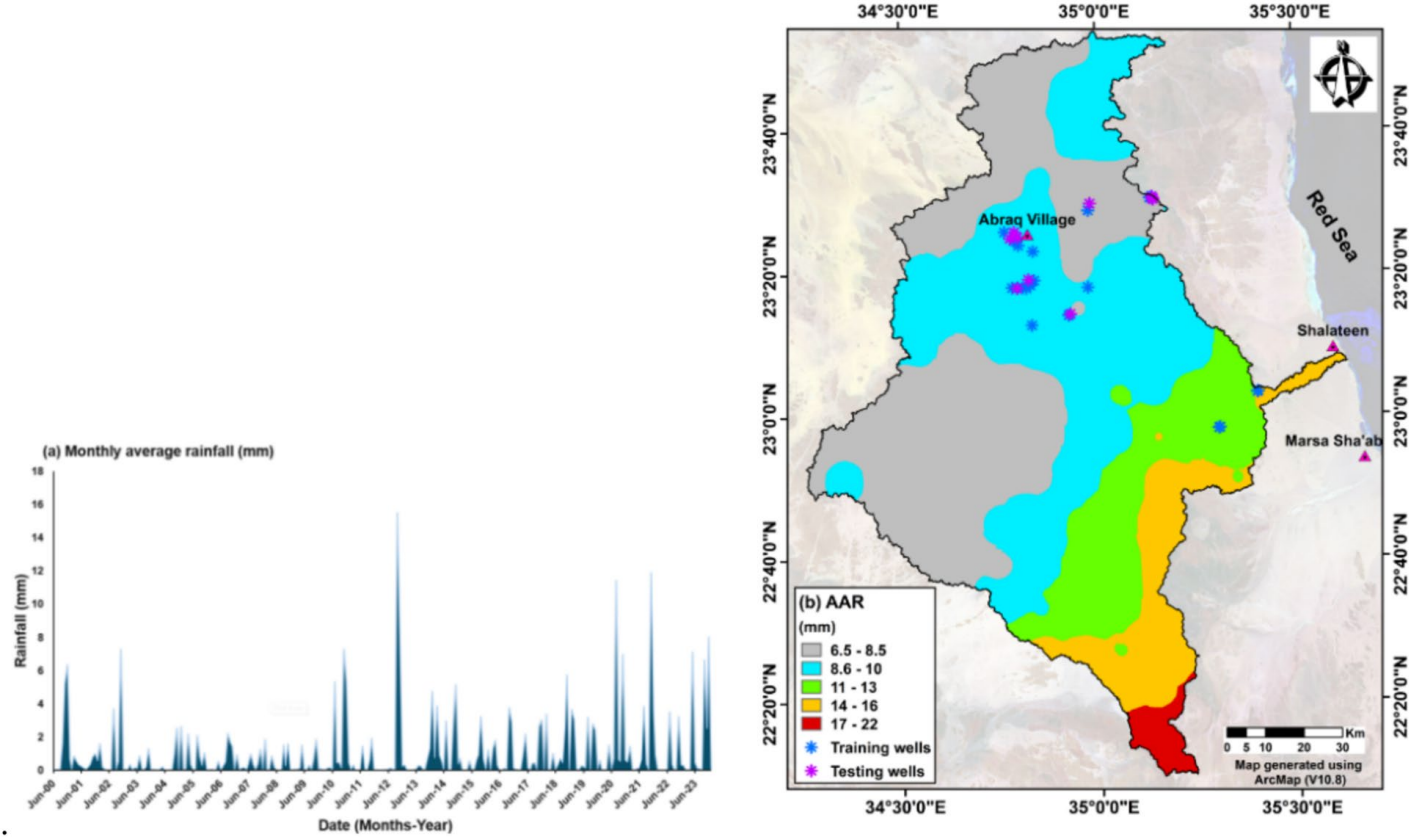
Climatic factor: (**a**) Monthly average rainfall (mm) between June 2000 and December 2023 using IMERG data for the study region, which is publicly available at the NASA Giovanni database https://giovanni.gsfc.nasa.gov/giovanni/; the figure is created using Microsoft Excel 365 V 2606 (https://www.microsoft.com/en/microsoft-365/Excel). (**b**) Average annual rainfall (AAR) map, which is obtained from the NASA Giovanni database https://giovanni.gsfc.nasa.gov/giovanni/; the figure is created using ArcGIS Pro v3.6 (https://www.esri.com/en-us/arcgis/products/arcgis-pro/overview).

Wadi Hodein is a major drainage basin located in the southeastern desert of Egypt, situated between latitudes 22°00’–24°00’ N and longitudes 34°00’–36°00’ E, covering an area of approximately 11,600 km^2^ (Fig. 1c). As a significant sub-catchment of the Red Sea mountains, the wadi functions as a primary conduit for surface runoff, channeling seasonal floodwaters eastward toward the Red Sea. The region is characterized by an arid climatic regime. Although annual precipitation is highly variable (Fig. 2), ranging from 3 mm to a maximum. 70 mm^48,50^. Intense winter thunderstorms over the adjacent highlands can occasionally generate 30 mm of rainfall within a single day. These episodic events frequently trigger severe flash floods that propagate through the wadi system. Relative humidity in the area fluctuates between 28 and 52%, reflecting the transitional nature of this coastal desert environment.

The Wadi Hodein basin is geographically defined by the Red Sea Mountain Range to the west and the town of Shalatein to the east (Fig. 1b,c). Despite the harsh aridity and hydrological variability, local Bedouin communities and residents depend almost exclusively.

## Geological and hydrogeological site characterization

**Fig. 3:**
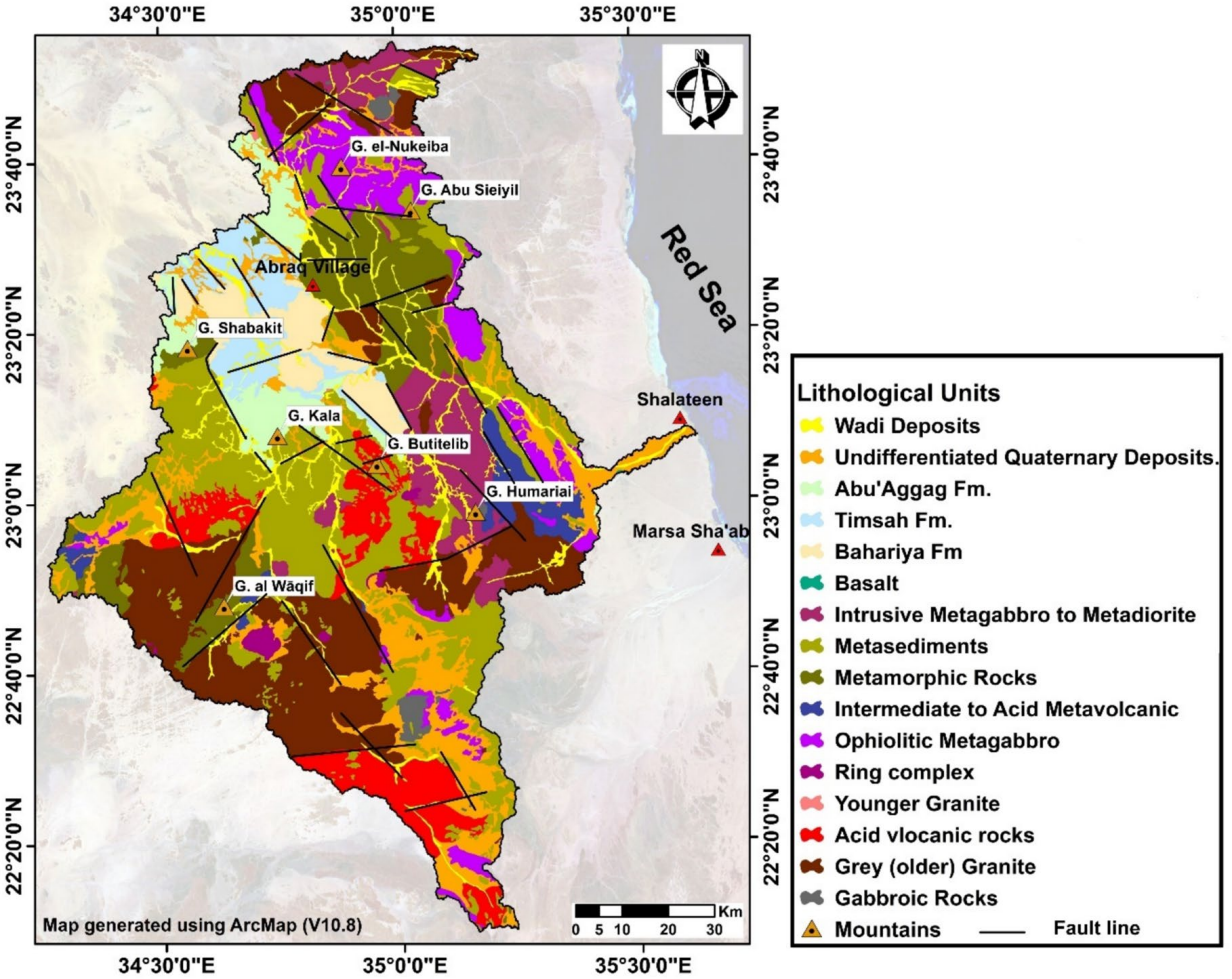
Geological Characterization of Wadi Hodein (a) Generalized geological map of the study area, which is obtained from^59^ via https://esdac.jrc.ec.europa.eu/content/geologic-map-egypt; the figure was prepared using ArcGIS Pro v3.6 (https://www.esri.com/en-us/arcgis/products/arcgis-pro/overview).

The geological setting of Wadi Hodein and its surroundings has been extensively explored in several studies (e.g., Hassan et al.^51^; Sadek et al.^52^; Sadek and Hassan^53^; Abdeen et al.^54^, Kusky and Ramadan^55^; Sadek^56^, El-kalioubi et al.^57^, Abd El-Wahed et al.^58^). As illustrated in the geological map (Fig. 3), the region is predominantly composed of complex crystalline rocks, Lower Cretaceous sandstones (NSAS), and Quaternary sediments^59^. The Late Proterozoic Precambrian basement rocks dominate large portions of the area, creating a landscape of high to medium relief characterized by weathered blocky mountains and hills in the western and southern regions (Fig. 3). This geological formation includes isolated hills of metasediments, metavolcanics, metagabbro complexes, older granitoids, acidic volcanics, and younger granitoids, along with tonalite–granodiorite belts^56–58^. In contrast, Cretaceous sandstones (NSAS) form thin beds that nonconformably overlie these basement rocks. Both the Precambrian and Cretaceous units have been intruded by Tertiary basalts associated with Red Sea rifting, further complicating the geological landscape.

The presence of NW–SE striking strike-slip and normal faults, as depicted on the geological map, plays a crucial role in shaping the geological units and influencing the distribution of the main drainage network in the area^48,49,57^. These fault systems facilitate the movement of groundwater by creating pathways for water flow and accumulation, thereby enhancing the connectivity between surface and subsurface water bodies.

The NSAS outcrops, represented by the Bahariya and Timsah formations, extend from Gebel Shabakit in the northwestern part to Gebel Butitelib in the central region of the basin (Fig. 3). In the eastern part of the area, Quaternary sandstone deposits overlie the basement complex, forming a continuous belt of outcrops across the Eastern Desert^50,59^. This region is intersected by numerous alluvial valleys that converge to create several main streams flowing toward the shoreline of the Red Sea.

Structurally, the dominant fault systems in the South Eastern Desert (SED) include the Meridional (N–S), Trans-African (NE–SW), Syrian Arc (ENE-WSW), Najd (WNW–ESE), Gulf of Suez-Red Sea (NW–SE), and Mediterranean Sea (E–W) trends^53,54,58^.

Hydrogeologically, the area features a unique hydrological system comprising three groundwater aquifers: Late Cretaceous Sandstone (NSAS), Quaternary deposits, and a fractured basement. The Cretaceous and Quaternary aquifers, which are disturbed by faults associated with dykes, represent the shallow source of water in the coastal plain of the SED^48,50,60^. The Nubian Sandstone aquifer is mainly composed of gravel, sand, and clay under confined conditions within the mountain valleys, which are recharged solely by sporadic rainfall^61^. The fractured basement aquifer, a shallow aquifer found in alluvial wadis, is an additional source of recharge^47^.

## Datasets and methodology framework

**Fig. 4:**
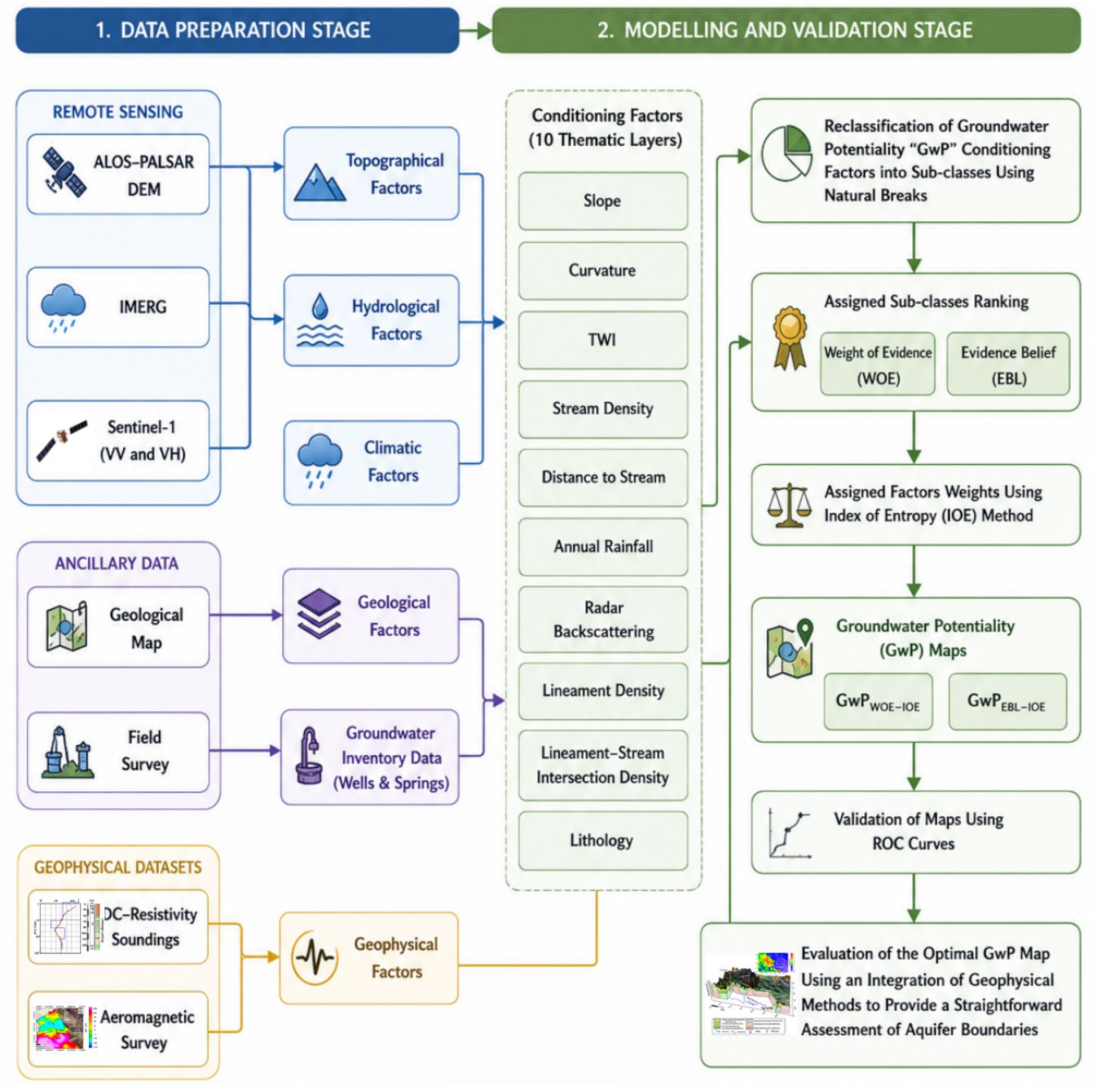
The Detailed methodology flowchart shows the integrative framework, including remote sensing and geophysical dataset analysis for enhancing the groundwater potential mapping.

Figure 4 illustrates the structured workflow of the integrated methodology applied in this study. The groundwater-potential (GwP) assessment combines topographic, geological, structural, hydrogeological, and climatic variables within a hybrid statistical framework. To further refine the analysis, the approach incorporates data from DC-resistivity soundings and aeromagnetic surveys. The subsequent sections describe each methodological step in detail.

### Datasets collection and preparation

The primary data sources include S1A VV and VH polarization imagery obtained from the Alaska Satellite Facility for lineament extraction and Rb extraction, the ALOS-DEM for topographic and hydrogeological parameter extraction, and IMERG data for rainfall distribution analysis. Additional datasets comprise groundwater well locations and a lithological map derived from the CONOCO^59^ database provided by the European Soil Data Center (https://esdac.jrc.ec.europa.eu/content/geologic-map-egypt). All digital datasets were geometrically corrected and co-registered to the Universal Transverse Mercator (UTM) coordinate system, WGS 1984 Zone 36N, using ArcGIS Pro 3.6^62^. The subsequent sections provide an in-depth analysis of the dependent inventory and independent factor datasets, emphasizing their significance within the modeling framework. Table 1 summarizes all datasets used in this study, including the source, acquisition date, native resolution, preprocessing steps, and the final coordinate reference system to which all layers were standardized.

**Table 1:** Summary of datasets used for thematic layer preparation.

Data type	Dataset	Date	Native specification & source	Preprocessing steps	Final coordinate reference system
Ancillary Data	Geological Map	1987	1:500,000 scale vector map. Source: CONOCO^59^; https://esdac.jrc.ec.europa.eu/	Digitized, georeferenced, converted from vector to raster (10 m cell size)	UTM Zone 36N, WGS84
Ancillary Data	Groundwater Wells	2021	Locations of 40 productive groundwater wells; Static water levels. Source: Field survey	GPS coordinates tabulated, imported as a point shapefile	UTM Zone 36N, WGS84
Remote Sensing	ALOS/PALSAR-DEM	11/06/2009	12.5 m spatial resolution; Radiometrically Terrain-Corrected (RTC) product; Fine Beam Single (FBS) polarization, HH. Source: Alaska Satellite Facility (ASF)^67^; https://search.asf.alaska.edu/	Twelve RTC scenes mosaicked; projected to UTM Zone 36N; used as base for slope, curvature, and hydrological extraction	UTM Zone 36N, WGS84
Remote Sensing	Sentinel-1A SAR	21/06/2021	Interferometric Wide (IW) mode; Ground Range Detected (GRD); VV + VH polarizations; 10 m × 10 m spatial resolution; Descending orbit. Source: Alaska Satellite Facility (ASF)	Orbit file application; thermal noise removal; radiometric calibration; Range-Doppler Terrain Correction; conversion to decibel (dB) scale; 3 × 3 Lee speckle filter	UTM Zone 36N, WGS84
Geophysical Data	Aeromagnetic Map	1980	Reduced-to-Pole (RTP) map. Source: Aero-Service^63^	Georeferenced; used for qualitative structural context only	N/A
Geophysical Data	VES Survey	Field Survey	17 Vertical Electrical Sounding points. Source: Field survey	Tabulated; used for qualitative subsurface validation only	N/A
Climate Data	GPM-IMERG Rainfall	Jan 1998–Jan 2019	Half-hourly estimates; 0.1° × 0.1° (~ 10 km) resolution. Source: NASA Giovanni^75^; https://giovanni.gsfc.nasa.gov/	Monthly aggregation; long-term annual average computed; interpolated to 10 m grid using Inverse Distance Weighting (IDW) with power parameter 2	UTM Zone 36N, WGS84

#### Potential controlling factors

Aly et al.^64^ emphasized that preparing thematic data layers and selecting relevant factors are critical components of any modeling approach. In this study, ten thematic parameters were identified to assess groundwater potential within the SCAq region, as they either influence or exhibit correlations with groundwater occurrence^8,24,48,50,60^. These parameters include slope, curvature, Topographic Wetness Index (TWI), Stream Density (SD), Distance to Stream (DTS), lithology, Radar backscattering (Rb), Lineament Density (LD), Lineament-Stream Intersection Density (LSID), and Average Annual Rainfall (AAR).

The selection criteria for these parameters are grounded in extensive literature that highlights their relevance to groundwater potential in the SCAq region, particularly in the context of the Eastern Desert’s complex terrain^50,60^. The regional geology features diverse lithological formations shaped by prevalent faults and fractures, as evidenced by the linear boundaries of rock units (Fig. 3). These geological characteristics play a crucial role in influencing groundwater storage and movement, affecting aquifer recharge dynamics.

Furthermore, hydrological factors such as Distance to Stream (DTS), Stream Density (SD), and the Topographic Wetness Index (TWI) are essential for identifying potential groundwater accumulation zones^65,66^. According to Sahour et al.^65^, areas in proximity to natural drainage systems within watersheds tend to exhibit shallower groundwater tables, which are critical for sustainable water resources. In this way, these parameters collectively inform our understanding of groundwater behavior and enhance the reliability of our modeling approach.

##### Topographic factors

Key topographic parameters, particularly slope and curvature, play a critical role in aquifer recharge, especially in the highly trained terrains of the Eastern Desert. These parameters were derived from an ALOS/PALSAR-1 Radiometrically Terrain-Corrected (RTC) digital elevation model (DEM) product, acquired on June 11, 2009, by the Alaska Satellite Facility (ASF)^67^. The dataset comprises twelve scenes in Fine Beam Single (FBS) polarization mode (HH), each with a native spatial resolution of 12.5 m. All scenes were downloaded in GeoTIFF format, mosaicked using the "Mosaic to New Raster" tool in ArcGIS Pro 3.6, and projected to the Universal Transverse Mercator (UTM) Zone 36N coordinate system with the World Geodetic System 1984 (WGS84) datum.

The spatial distribution of surface slope was calculated using the Slope tool (ArcGIS Pro V3.6), which calculates the maximum rate of change in elevation between each cell and its eight neighbors. The output slope grid was computed in degrees. This parameter is essential for understanding how water moves across the landscape. Additionally, the Curvature tool was utilized to assess deviations from a planar surface, generating an output grid where negative values represent concave, positive values represent convex, and zero values represent flat surfaces. These topographical features significantly influence hydrological processes, determining areas of water accumulation and influencing the efficiency of aquifer recharge.

##### Hydrological factors

Hydrological factors, including DTS, SD, and TWI, offer critical insights into potential groundwater accumulation zones^66^. Consequently, the mosaicked 12.5 m ALOS-DEM was used as input for hydrological analysis. Areas in proximity to natural drainage systems within watersheds are more likely to exhibit a shallow groundwater table^65^. Consequently, ALOS-DEM data were used to extract the natural drainage network maps. The spatial distribution of stream networks was obtained by applying the eight-direction (D-8) flow algorithm^68^ using the Arc Hydro toolset (version 2.0) in ArcMap. The stream network was delineated using a flow-accumulation threshold of 2000 pixels, such that only cells draining at least 2000 upstream cells were included in the network. The SD, defined as the ratio of total stream length to drainage basin area, was derived using the Line Density tool, with a search radius of 1000 m and an output cell size of 10 m. The TWI is widely used to assess soil moisture distribution and saturation zones within a given terrain^69^. The TWI was computed using the following Eq. (1), implemented in the ArcGIS Raster Calculator.

1 $$\mathrm{T}\mathrm{W}\mathrm{I}= \mathrm{l}\mathrm{n}\left(\frac{a}{\mathrm{tan}\mathrm{b}}\right)$$

where (a) is the local upslope contributing area (m^2^/m), (b) is the slope gradient (measured in degrees) at that specific position, and “ln” is a constant that refers to the Napierian logarithm.

##### Geological factors

The variations in surface geology and soil distribution directly affect hydrological processes, determining how effectively water infiltrates into the ground and replenishes aquifers. Each rock type possesses distinct properties that significantly influence surface water movement and soil porosity within the basins. The lithological map used in this analysis was derived from the published geological map of CONOCO^59^, scaled at 1:500,000 (Fig. 3). The geological map was scanned, georeferenced in ArcGIS using ground control points, and the geological polygons were digitized. This thematic vector layer was then converted to raster format at a 10 m cell size using the Polygon to Raster tool to standardize the layer, facilitating the assignment of weights and ranks.

Lineaments, observed as linear or curvilinear features in satellite imagery, encompass geological structures such as faults, fractures, and joints, playing a crucial role in groundwater flow and accumulation^27^. Two high-resolution Ground Range Detected (GRD) scenes from S1A VV and VH polarizations, acquired in June 2021 using the Interferometric Wide (IW) mode, were used due to the availability of the images over the whole basin at no cost (Source of data). These images were captured in a descending orbit with a spatial resolution of 10 m × 10 m. The preprocessing included applying the orbit file, speckle filtering, radiometric calibration, thermal noise removal, and Range-Doppler Terrain Correction. The images were subsequently converted to a decibel scale to mitigate disturbances in the VV and VH bands. A 3 × 3 Lee filter was applied to minimize speckle noise while preserving structural integrity, particularly in homogeneous regions and along feature boundaries^70,71^.

Lineaments, observed as linear or curvilinear features in satellite imagery, encompass geological structures such as faults, fractures, and joints^27^. For their extraction, two high-resolution Ground Range Detected (GRD) scenes from Sentinel-1A (S1A) with VV and VH polarizations were acquired. The scenes, captured on June 21, 2021, in Interferometric Wide (IW) mode with a descending orbit, were downloaded from the Alaska Satellite Facility (ASF) vertex portal. Each scene had a native spatial resolution of 10 m × 10 m. Preprocessing was performed using the Sentinel-1 Toolbox in SNAP (ESA SNAP 9.0) and included: (1) Apply Orbit File, (2) Thermal Noise Removal, (3) Radiometric Calibration to sigma0, (4) Range-Doppler Terrain Correction using the SRTM 30 m DEM, and (5) Conversion to decibel (dB) scale. To mitigate speckle noise while preserving structural integrity, a 3 × 3 Lee filter was applied to the calibrated dB images^70,71^.

Lineament extraction was performed using the LINE module in CATALYST PROFESSIONAL PCI Geomatica 2022 (version 23.0). The filtered VV polarization dB image was used as input. The following parameter values were set for the extraction algorithm, with GTHR and ATHR adjusted from default values to enhance geological feature detection, following Embaby et al.^8^

The extracted vector lineaments were manually reviewed and edited to remove non-geological features (e.g., roads, field boundaries). The Intersect tool in ArcGIS was subsequently applied to determine intersection points between the extracted lineaments and stream networks. Structural indices, including LD and LSID, were computed using the Kernel Density tool in ArcGIS Pro V3.6 with a search radius of 1000 m and an output cell size of 10 m.

Radar backscatter intensity has been widely recognized as an effective parameter for monitoring hydrological components^72,73^. A static regional Rb image was generated from the preprocessed S1A VH polarization dB image. The image was resampled to a 10 m grid to analyze spatial variations in soil properties.

##### Climatic factors (average annual rainfall)

Rainfall serves as the primary water source in arid and semi-arid basins^74^. Daily precipitation data were acquired from the NASA Global Precipitation Measurement (GPM) mission’s Integrated Multi-satellitE Retrievals for GPM (IMERG) Final Run product (Version 06). The data were accessed via the NASA Giovanni platform (https://giovanni.gsfc.nasa.gov/giovanni/) for the period from January 1, 1998, to January 31, 2019^75^. This dataset provides half-hourly precipitation estimates at a native spatial resolution of 0.1° × 0.1° (approximately 10 km × 10 km). The daily data were aggregated to monthly, then to annual totals. A long-term Average Annual Rainfall (AAR) grid was computed by averaging the 21 annual grids. This coarse AAR raster was then projected to UTM Zone 36N, WGS84, and interpolated to a 10 m grid cell size to match other thematic layers using the Inverse Distance Weighting (IDW) geostatistical method in ArcGIS, with a power parameter of 2 and a variable search radius. AAR was identified as a relevant conditioning factor, given its applicability over extensive spatial scales^75^.

#### Groundwater inventory data

The residents utilize springs, where natural groundwater is discharged from the NSAS^49^, along with drilled groundwater wells for daily use and agricultural activities^45^. A total of 40 productive groundwater (PGw) points, including productive well sites and a few spring locations, were identified using Google Earth Pro imagery and field observations within the study area. To satisfy the presence/absence requirements of the bivariate models and improve data distribution, absence points were generated from two complementary sources: (1) documented dry wells (shallow, non-productive boreholes) encountered in the field, and (2) bedrock exposures and high-terrain regions identified from satellite imagery and geological maps, which are characterized by high runoff and minimal overburden, making them inherently unfavorable for groundwater storage. To account for the influence of local faults and aquifer discontinuities, a 100-m buffer zone was established around all presence points. No absence points were sampled within these buffers to prevent conflict with productive structural areas.

For modeling, 28 PGw points (70%) and an equivalent number of absence points were randomly selected for training the WOE and EBF models, while the remaining 12 PGw points (30%) were reserved for validation. Final independent validation was performed using 17 Vertical Electrical Sounding (VES) points, which were excluded from the training process entirely.

### Hybrid GIS-based bivariate analysis for groundwater potential mapping

Selecting an appropriate decision rule is crucial, as it determines the ranking and weighting methodology used to integrate the influencing factors under predefined weights, thereby significantly affecting the analysis outcomes. This study employed two bivariate geospatial models, Weight of Evidence (WoE) and Evidential Belief Function (EBF), to evaluate the ranking of sub-class factors within the investigated basin.

All raster layers, except for lithology and curvature, were reclassified into five scoring levels using the natural breaks (Jenks) classification method, which operates at a spatial resolution of 12.5 m. This classification approach enhances differentiation among classes by identifying optimal threshold values that group similar data points based on inherent patterns. The resulting framework effectively represents the diverse topographic, geological, structural, hydrogeological, and climatic characteristics of the study area, each of which plays a role in influencing the spatial distribution of groundwater potential (GwP) within SCAq.

To examine the intricate relationships between dependent and independent controlling factors, 70% of the groundwater inventory points were utilized. The Index of Entropy (IOE) method was deployed to assign weights to the conditioning factors affecting GwP. Furthermore, a hybrid modeling framework was developed by integrating IOE with WoE and the EBF method to generate predictive GwP maps.

The resulting groundwater potential models were subsequently validated using the Area Under the Curve (AUC) approach, with the remaining 30% of the dataset employed for model reliability assessment. These models were developed using the Spatial Data Modeller (SDM) extension in ArcMap 10.8, along with XLSTAT and IBM SPSS Statistics. A comprehensive description of the adopted methodology is provided in the subsequent sections.

#### Index of entropy (IOE) model

The Index of Entropy (IOE) method is commonly employed to assess the uncertainty and instability of a system^76^. In this study, the IOE was utilized to determine the predictive weights (${\mathrm{W}}_{\mathrm{j}}$) of each controlling factor based on the following equations^76^.

2 $$Pij=FR=\frac{\raisebox{1ex}{$A$}\!\left/ \!\raisebox{-1ex}{$B$}\right.}{\raisebox{1ex}{$C$}\!\left/ \!\raisebox{-1ex}{$D$}\right.}=\frac{b}{a}$$

3 $$(P\mathrm{i}\mathrm{j})=\frac{Pij}{\sum_{i=1}^{sj}Pij}$$

4 $$\mathrm{H}\mathrm{j}=-\sum_{i=1}^{Sj}\left(Pij\right)\mathrm{log}\left(Pi., nj\right), j=1, \dots , n.$$

5 $$H_{j\max } = \log_{2} Sj$$

6 $$\mathrm{I}j=\frac{H jmax -Hj}{H jmax} I=(\mathrm{0,1}) j= 1, \dots ,n$$

7 $$w_{j} = I_{j} P_{ij}$$

where the frequency ratio (FR) quantifies the spatial association between a specific factor class and the target phenomenon. For a given groundwater factor, (A) represents the area of a specific class within that factor, while (B) is the total area of the entire factor. Similarly, (C) denotes the number of pixels in that class area, and (D) is the total number of pixels in the study area. The term (b) is the percentage area of a specific class relative to the factor’s total area, whereas (a) is the percentage area of the factor relative to the entire study domain. The probability density is denoted by Pij. Entropy values are expressed as H_j_ (actual entropy for factor j) and H_jmax_ (maximum possible entropy for that factor). S_j_ is the number of classes within factor j, I_j_ is the information coefficient reflecting the factor’s predictive power, and w_j_ is the resultant weight value assigned to the factor as a whole, which ranges between 0 and 1.

GWP map of the entropy method was prepared by overlaying all the thematic layers in terms of the obtained weights of each model through the spatial analysis tool in the ArcGIS package using the following equation:

8 $$\mathrm{G}\mathrm{W}\mathrm{P}=\sum_{i=1}^{n}Wi*Ri$$

where GWP is the groundwater potential zone; Wi is each layer weight and Ri the class rates within a thematic layer.

#### Weight of evidence (WOE) model

The Weight of Evidence (WoE) model is a data-driven, Bayesian method that estimates the probability of groundwater occurrence given a specific evidential class. For each class, it computes two weights: a positive weight (W^+^) when the class is present and a negative weight (W^−^) when it is absent. The contrast, C = W^+^ − W^−^, summarizes the class’s overall spatial association with groundwater; larger positive contrasts indicate a stronger favorable relationship.

These weights are derived by comparing the density of presence points (groundwater occurrences) within a class to the density of absence points outside that class. As a bivariate statistical technique, WoE combines simple logical ratios with Bayesian probability to quantify the evidence provided by each factor class for predicting groundwater presence^77^. This produces objective, class-level weights that reduce subjective bias and help reveal complex interdependencies among conditioning factors^78^. The positive and negative weights for each sub-category are computed using the following equations^79,80^.

9 $${W}^{+}=\mathrm{ln}\frac{P\left(\frac{F}{G}\right)}{P\left(\frac{F}{\overline{G} }\right)}$$

10 $${W}^{-}=\mathrm{ln}\frac{P\left(\frac{\overline{\mathrm{F}}}{G }\right)}{P\left(\frac{\overline{\mathrm{F}} }{\overline{G} }\right)}$$

where F and $\overline{\mathrm{F} }$ represent the presence and absence of conditioning parameters, respectively; P denotes probability; and G, and $\overline{\mathrm{G} }$ correspond to the presence and absence of groundwater, respectively.

Standard deviation of *W* is calculated as follows^80^:

11 $$S\left(c\right)= \sqrt{{S}^{2}{W}^{+}+{S}^{2 }}{W}^{-}$$

where *S*^2^*W*^+^ and *S*^2^*W*^−^ are defined as the variances of positive weights and negative weights, respectively.

The final WOE coefficients (${R}_{WOE}$) assigned to each factor class can be obtained as follows.

12 $${R}_{WOE}=\left({W}^{+}\right)+\left({W}_{min}^{-}\right)-\left({W}^{-}\right)$$

where ${W}_{min}^{-}$ represents an overall measure of spatial association between the training points and the evidential theme, combining the effects of the two weights. Sometimes, W^+^ can be close to zero, yet W^-^ is strongly negative. This situation arises if the presence of the theme is not particularly predictive of training points, but the absence of the theme provides strong evidence that points are unlikely to occur. Conversely, there can be an imbalance between the absolute values of W^+^ and W^-^ in the other direction, or the two weights can have absolute values in about the same range.

According to Bonham-Carter^80^, the absolute values of weights provide a measure of predictive strength: values between 0 and 0.5 are mildly predictive; values between 0.5 and 1 are moderately predictive; values between 1 and 2 are strongly predictive; and values greater than 2 are extremely predictive.

The final ${GwP}_{\mathrm{W}\mathrm{O}\mathrm{E}-\mathrm{I}\mathrm{O}\mathrm{E}}$ map was generated by applying the inferred IOE weight (${\mathrm{W}}_{j}$) to the ranked numerical values of factor classes, represented as attributes of ${R}_{WOE}$.

13 $${GwP}_{\mathrm{W}\mathrm{O}\mathrm{E}-\mathrm{I}\mathrm{O}\mathrm{E}}= \sum {\mathrm{W}}_{j}\times {\mathrm{R}}_{WOE}$$

In standard WOE, classes with zero presence or zero absence points yield infinite or undefined weights, causing numerical instability. In the study area, for example, the basement bedrock lithological class contained zero productive groundwater points compared to the Nubian Sandstone and Alluvium deposits. To address this, a correction factor of 0.5 was added to all presence and absence counts following Bonham-Carter^80^, using the ArcSDM toolbox. This correction yielded a finite negative weight (W^+^ = − 1.25) for the basement bedrock class, correctly indicating unfavorable conditions while preserving numerical stability.

##### Assessment of predictor independence

A fundamental assumption of the Weight of Evidence model is that evidential themes are conditionally independent with respect to the training points^80^. Violation of this assumption can lead to overestimation of posterior probabilities and unstable weight estimates^81^. To evaluate the degree of dependence among the ten conditioning factors, a multicollinearity assessment was conducted using pairwise Pearson correlation and Variance Inflation Factor (VIF).

Pearson correlation coefficients (r) were calculated between all factor pairs (Table S1), with |r|> 0.7 indicating a strong correlation^82^. VIF was computed as VIF = 1/(1 − R^2^), where values exceeding 5 indicate moderate multicollinearity concerns (Table S2)^83^. Strong correlations were observed between physically coupled pairs: Slope–TWI (r = − 0.82), LD–LSID (r = 0.86), DTS–SD (r = 0.71), and Slope–Rb (r = 0.73). VIF values remained within acceptable ranges (< 5) for most factors, with LSID showing a slightly elevated value (5.26) due to its correlation with LD. Curvature and Rainfall exhibited weak correlations with all other factors (VIF < 1.3), confirming their independent contributions. Despite these correlations, all factors were retained due to their conceptual distinctiveness: Slope controls runoff velocity while TWI identifies saturation zones; LD reflects fracture intensity while LSID specifically targets permeable fracture intersections^82,83^

These correlations reflect the inherent physical relationships among the factors rather than data artifacts. Although all factors were retained due to their conceptual distinctiveness, WOE is known to be sensitive to conditional dependence. To address this, the ten factors were organized into five conceptual groups based on their hydrogeological roles: topographic (Slope, TWI, Curvature), structural (LD, LSID), hydrologic (DTS, SD, Rainfall), geologic (Lithology), and surface (Radar backscattering). Within each group, moderate to strong correlations are expected because the factors capture different dimensions of the same subsurface process. For example, LD measures the frequency of lineaments, while LSID targets their intersections. Together, their combined contribution more effectively represents structural control on groundwater flow than either factor alone. Rather than treating correlated factors as independent predictors, this grouping framework interprets their joint contribution as reflecting an integrated hydrogeological process^82,83^.

#### Evidential belief function (EBF) model

The Evidential Belief Function (EBF) is a knowledge-driven spatial modeling framework used here to assess groundwater potential by integrating multiple conditioning factors^84^. In this approach, thematic layers representing factors that control groundwater occurrence (e.g., lithology, slope, land use) are treated as distinct pieces of evidence. The model then synthesizes this evidence to produce predictive groundwater potential maps^80^. This synthesis is achieved by quantifying the spatial correlation between each evidential layer and the locations of existing groundwater wells. By evaluating how strongly each factor class is associated with well presence, the model derives weights that form the basis for the final integration. This data-driven correlation enhances the objectivity and reliability of the predictive map.

A key advantage of the EBF model is its capacity to not only delineate prospective groundwater zones but also to explicitly quantify the uncertainty inherent in those predictions^84^. This dual output is particularly valuable for decision-making, as it provides stakeholders with a measure of confidence in the mapped zones, thereby supporting more nuanced and effective groundwater management strategies.

The model is formalized within the Dempster-Shafer theory of evidence, which generalizes traditional Bayesian probabilities into lower and upper probability bounds^85^. In this framework, the lower and upper probabilities correspond to the belief (Bel) and plausibility (Pls) measures, respectively. The framework also explicitly quantifies related measures, including the degree of disbelief (Dis), which represents support against a proposition, and the degree of uncertainty (Unc), which reflects a lack of knowledge or conflict between pieces of evidence^86^. Together, these four functions (Bel, Dis, Unc, and Pls) provide a comprehensive representation of the evidential support for groundwater occurrence across the study area.

A detailed explanation of the algorithm is provided by Carranza et al.^85^. In the context of groundwater potential mapping using EBF^86,87^, the frame of discernment is defined by Eqs. (14) and (15):

14 $$\uplambda \left(\mathrm{T}\mathrm{p}\right){\mathrm{E}}_{\mathrm{i}\mathrm{j}}= \frac{\left[\frac{\mathrm{N}\left(\mathrm{G}\cap \mathrm{E}\mathrm{i}\mathrm{j}\right)}{\mathrm{N}\left(\mathrm{G}\right)}\right]}{[\frac{\mathrm{N}\left({\mathrm{E}}_{\mathrm{i}\mathrm{j}}\right)-\mathrm{N}\left(\mathrm{G}\cap \mathrm{E}\mathrm{i}\mathrm{j}\right)}{\mathrm{N}\left(\mathrm{C}\right)-\mathrm{N}(\mathrm{G})}]}$$

where:

λ(Tp)E_ij = Weight of evidence for the presence of groundwater given the class (j) of factor (i). This is a dimensionless ratio.

N (G ∩ E_ij) = Number of pixels containing groundwater wells that also fall within class (j) of factor (i).

N(G) = Total number of groundwater well pixels in the entire study area.

N(E_ij) = Total number of pixels comprising class (j) of factor (i).

N(C) = Total number of pixels in the entire study area.

The belief measure is given by:

15 $$\mathrm{B}\mathrm{e}\mathrm{l}=\frac{\uplambda \left(\mathrm{T}\mathrm{p}\right){\mathrm{E}}_{\mathrm{i}\mathrm{j}}}{\sum\uplambda \left(\mathrm{T}\mathrm{p}\right){\mathrm{E}}_{\mathrm{i}\mathrm{j}}}$$

The degree of disbelief is defined as:

16 $$\mathrm{D}\mathrm{i}\mathrm{s}=\frac{\uplambda \left(\overline{\mathrm{T}\mathrm{p} }\right){\mathrm{E}}_{\mathrm{i}\mathrm{j}}}{\sum\uplambda \left(\overline{\mathrm{T}\mathrm{p} }\right){\mathrm{E}}_{\mathrm{i}\mathrm{j}}}= \frac{\left[\frac{\mathrm{N}\left(\mathrm{G}\right)-\mathrm{N}\left(\mathrm{G}\cap \mathrm{E}\mathrm{i}\mathrm{j}\right)}{\mathrm{N}\left(\mathrm{G}\right)}\right]}{[\frac{\mathrm{N}\left(\mathrm{C}\right)-\mathrm{N}\left(\mathrm{G}\right)-\mathrm{N}\left({\mathrm{E}}_{\mathrm{i}\mathrm{j}}\right)+\mathrm{N}\left(\mathrm{G}\cap \mathrm{E}\mathrm{i}\mathrm{j}\right)}{\mathrm{N}\left(\mathrm{C}\right)}]}$$

Uncertainty is calculated as:

17 $$\mathrm{U}\mathrm{n}\mathrm{c}=[1-\mathrm{B}\mathrm{e}\mathrm{l}-\mathrm{D}\mathrm{i}\mathrm{s}]$$

Plausibility (Pls, representing the maximum possible support for groundwater occurrence. It is the sum of belief and uncertainty, and is dimensionless. Pls is expressed as:

18 $$\mathrm{P}\mathrm{l}\mathrm{s}= [1-\mathrm{D}\mathrm{i}\mathrm{s}]$$

The final GwP map generated using the EBF-IOE model was obtained by applying the inferred IOE weight (Wj) to the ranked numerical values of factor classes represented as attributes of Bel:

19 $${GwP}_{\mathrm{E}\mathrm{B}\mathrm{F}-\mathrm{I}\mathrm{O}\mathrm{E}}= \sum {\mathrm{W}}_{j}\times Bel$$

The GwP maps were classified into five susceptibility levels: very low, low, moderate, high, and very high, employing the natural breaks classification method in ArcMap.

### Validation of groundwater potential (GwP) models

The validation of GwP models was conducted using a combination of statistical, spatial, and field-based approaches to ensure robust evaluation of predictive performance and generalizability. Given the relatively small dataset and the influence of spatial autocorrelation, multiple complementary validation techniques were employed.

A repeated k-fold cross-validation (CV) framework was implemented to overcome the limitations of a single random data split. The dataset, comprising 40 productive groundwater points and 40 absence points (n = 80), was subjected to 5 iterations of fivefold CV. In each iteration, the data were randomly divided into five subsets, with four folds (64 points) used for training and the remaining fold (16 points) used for testing. This repeated resampling generated a distribution of performance metrics, allowing the computation of mean Area Under the Curve (AUC) values and corresponding standard deviations, thereby providing a reliable estimate of model accuracy and associated uncertainty.

To further address the effects of spatial autocorrelation, spatially blocked cross-validation was applied as the primary test of model generalization. The study area was divided into five spatially contiguous blocks based on natural geographic boundaries. Models were iteratively trained on four blocks and evaluated on the remaining block until each block had been used as a test set. Importantly, the spatial blocks used for validation were defined independently of the groundwater occurrence clustering employed during model development. The blocked cross-validation was applied only during model evaluation and did not influence the generation of training samples or the spatial distribution of productive and non-productive groundwater points. Consequently, the blocked validation provides an independent assessment of model generalization capability in previously unsampled areas and offers a more conservative estimate of predictive performance than conventional random cross-validation.

Model discrimination ability was evaluated using the Receiver Operating Characteristic (ROC) curve analysis^30,88^. For each validation iteration, ROC curves were generated by plotting the True Positive Rate (TPR; sensitivity) against the False Positive Rate (FPR; 1 − specificity) across a range of classification thresholds. The Area Under the ROC Curve (AUC) was calculated using the trapezoidal rule^85,86^:

20 $$\mathrm{A}\mathrm{U}\mathrm{C}=\frac{\sum \left[\mathrm{T}\mathrm{R}\mathrm{P}\left(\mathrm{i}+1\right)+\mathrm{T}\mathrm{R}\mathrm{P}(\mathrm{i})\right]}{2}\times \left[\mathrm{F}\mathrm{P}\mathrm{R}\left(\mathrm{i}+1\right)-\mathrm{F}\mathrm{P}\mathrm{R}(\mathrm{i})\right]$$

where *TPRi* and *TPRi* + 1 represent the true positive rates, and *FPRi* and *FPRi* + 1 represent the false positive rates at consecutive threshold values (*i*) and (*i* + *1)*. Final reported AUC values represent the mean across all CV iterations with associated standard deviation. AUC values range from 0 to 1; values above 0.7 are considered acceptable, and those above 0.9 indicate outstanding predictive accuracy^88^.

In addition to statistical validation, frequency ratio analysis was employed to assess the spatial agreement between observed groundwater occurrences and predicted suitability classes. This evaluation was based on two assumptions: (i) high and very high GwP zones should occupy a relatively smaller proportion of the study area, and (ii) a reliable model should assign the majority (> 50%) of observed groundwater points to at least moderate suitability zones, with higher concentrations in high and very high classes. This spatial validation provides an intuitive measure of model consistency with observed patterns.

Finally, independent ground truth validation was conducted using 17 VES points and field observations from existing wells and springs located within high and very high potential zones. These VES data were not included in model training, ensuring independence of validation. The geophysical data provided critical insights into subsurface conditions, including lithological variations, aquifer thickness, depth to bedrock, groundwater levels, salinity, and structural features such as faults and fractures.

To further support model validation, detailed geophysical investigations were carried out in high and very high GwP zones of Wadi Hodein. The integration of aeromagnetic data and VES measurements enabled the identification of subsurface lithological variations and structurally controlled aquifer systems. Aeromagnetic data delineated faults and fractures, while VES data characterized resistivity variations associated with groundwater saturation and salinity. The strong agreement between geophysical evidence and predicted high-potential zones confirms the reliability of the GwP models in identifying structurally controlled groundwater systems.

### Geophysical investigations

**Fig. 5:**
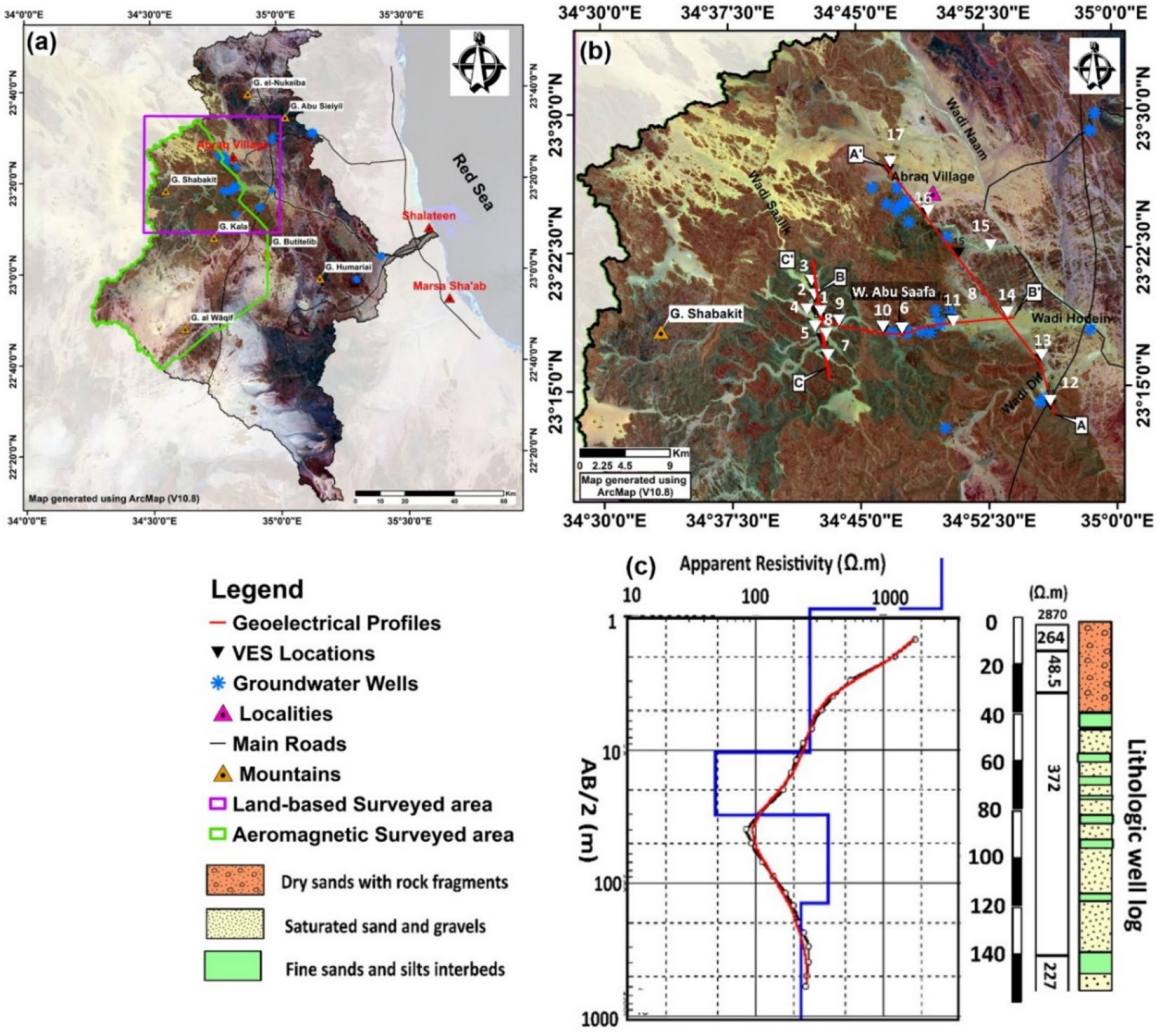
Geophysical Datasets in the Identified High Potential Zone: (a) Aeromagnetic survey area and locations of land-based Vertical Electrical Sounding (VES); (b) A zoomed-in map of VES points, geoelectrical cross-sections, and drilled wells; (c) Calibration of resistivity interpretation of VES12 alongside the lithologic log of a drilled well.at Wadi Dif. These figures were prepared using ArcGIS Pro v3.6 (https://www.esri.com/en-us/arcgis/products/arcgis-pro/overview). The VES curve interpretation was generated using IPI2win free software available at http://geophys01.geol.msu.ru/ipi2win.htm.

Detailed geophysical investigations were conducted within the high and very high GwP and the surrounding zones of Wadi Hodein to assess the role of subsurface structures in controlling aquifer systems (Fig. 5a). Drilled wells and natural springs show significant variability in depth, yield, and water quality over short distances, reflecting the complexity of the subsurface and the presence of diverse aquifer systems.

A DC resistivity survey (17 VESs) was intentionally targeted within these zones to address subsurface complexities, specifically fault impacts and basement relief, that remote sensing and WOE modeling cannot fully capture. Low-potential zones were not surveyed due to logistical constraints and the focused goal of validating high-potential targets.

To overcome the surface limitations of remote sensing and RadarSat, aeromagnetic and VES data were integrated. Aeromagnetic data delineated lithological variations and structural features (faults and fractures), while VES provided resistivity profiles to identify aquifer boundaries, saturation, and water salinity. Together, these geophysical approaches validated and strengthened the understanding of structurally controlled groundwater systems in the study area.

#### Processing of aeromagnetic data

Magnetic anomalies occur due to the uneven distribution of magnetic minerals in the Earth’s upper crust, which leads to variations in the magnetic field. The spatial patterns and distribution of these anomalies offer valuable insights into the presence of magnetite-bearing rock formations and the depth of the basement surface^19^. This information helps clarify the underlying subsurface hydro-structures^18^.

The original airborne magnetic survey was conducted through a collaborative effort between the Egyptian government and the Aero-Service Division of Western Geophysical Company, USA^63^. Flight lines were flown in the NE-SW direction with 1.5 km spacing, while tie lines were oriented NW–SE with 10 km spacing, at a nominal flying altitude of 120 m above ground level^63^. Preprocessing included diurnal variation correction, International Geomagnetic Reference Field (IGRF) removal (IGRF 1975), and micro-leveling using standard tie-line adjustment procedures^63^.

The Reduced-to-Pole (RTP) transformation was applied to compensate for the Earth’s magnetic field inclination, relocating anomaly maxima directly above their causative sources^89^. Using Geosoft Oasis Montaje V. 8.3.3, the RTP was computed with an inclination angle of 35.5° and a declination angle of 2.5°, applied to the gridded data (100 m cell size) via a wavenumber domain filter.

The Tilt Angle Derivative (TAD), an edge detection technique, was employed to delineate subsurface magnetic source boundaries, with fault locations identified from zero-value contours^90^. The TAD was calculated from the RTP grid using a 3 × 3 convolution kernel in Geosoft Oasis V. 8.4. Additionally, the Analytical Signal (AS) technique was utilized to enhance near-surface features, with the AS grid filtered using a 3 × 3 median filter to reduce noise while preserving structural boundaries.

#### Vertical electrical sounding (VES)

DC resistivity surveys are widely used in groundwater exploration to delineate aquifer geometry, lithological variations, and fracture networks via resistivity contrasts^91^. When integrated with remote sensing-derived lineament density and surface structural data, they better characterize recharge systems by linking surface lineaments with subsurface conductive zones and improving 3D aquifer modeling^92^.

At the local scale, a Schlumberger DC resistivity survey was conducted to delineate potential groundwater zones by analyzing vertical resistivity variations. Seventeen VES points were measured using a four-electrode Schlumberger configuration with a maximum electrode spacing of 1000 m, enabling investigation of subsurface layers to depths of about 200–250 m for groundwater potential assessment (Fig. 5b). These soundings were acquired along three profiles to assess how subsurface structures and geology influence the aquifer within the identified high groundwater potential (GwP) zone. The survey was also designed to improve the lateral mapping of the Nubian Sandstone (NSAS), especially along the fault zone where natural springs occur between basement rocks and the NSAS. All 17 VES soundings were collected before the WoE and EBF modeling, and were positioned within the NSAS and adjacent basement exposures to support the conceptual hydrogeological model and evaluate structural controls on groundwater distribution.

These profiles were defined using spatial analysis of lineament density and drainage patterns, field-observed lithological variations, and data from available boreholes and springs. Profile AA’ (NW–SE) connects the Abraq spring to the Wadi Dif well, crossing Wadi Hodein and multiple east–west lineaments. Profile BB’ (E–W) follows Wadi Hodein through Wadi Abu Saafa, where variations in drilled-well depth and discharge indicate the role of basement relief and faults. Profile CC’ (N–S) links upstream drainages of Wadi Abu Saafa, supporting recharge along Wadi Abu Saafa and Wadi Hodein.

The sounding curves were inverted using a 1D Newton-based inversion scheme in IPi2win Ver. 3.0 software^93^. To reduce non-uniqueness (equivalence), the inversion was constrained using nearby boreholes: each profile contains at least one well, and its lithology was used to build the initial reference model near each VES, guiding the layer number, initial resistivities, and water-table depth from observed stratigraphy. The RMS% was limited to 3–5%, and the resulting uncertainty was used to estimate bounds on interpreted resistivity and thickness for each VES.

Finally, geoelectrical results were calibrated with lithological information from well logs (Fig. 5b), enabling mapping of vertical and lateral variations in true subsurface resistivity and associated structures (Fig. 5c). Figure S1 (Supplementary) presents representative VES points and their geological interpretations.

## Results

### Comparative analysis of factors affecting groundwater potential

The variety in geological and hydrogeological features within the Wadi Hodein basin plays a critical role in determining groundwater potential. A geostatistical analysis was performed to identify potential groundwater locations by examining the relationships among geomorphological, lithological, structural, hydrogeological, and climatic factors, along with training groundwater potential (GwP) data from the area. Table 2 presents the results of the statistical analysis, including the EBF and WOE ranking values for each subclass layer, as well as the factor weight values obtained through IOE. If a specific class exhibits no groundwater occurrence (Table 2), the probability of assigning a corresponding belief value is negligible or effectively zero (Bel = 0). Conversely, BEL values greater than zero indicate a significant association between the respective class and groundwater occurrence^94^. Furthermore, positive WOE values indicate a favorable association with groundwater occurrence, whereas negative values suggest an unfavorable relationship, thereby quantifying the spatial correlation between causative factors and groundwater occurrence potential (Table 2).

**Table 2:** Bivariate statistics of weighting and ranking values for controlling factors based on their relative importance on groundwater potentiality (GwP).

Factor	Class range	Class area (%)	Percentage of wells (%)	W+	W−	W_f_	Bel	Dis	Unc	Pls	Pij	Ej	1-Ej	Wj
Slope (°)	> 27	44.739	32.47	− 0.32	0.20	− 0.45	0.084	0.278	0.638	0.722	0.08	− 0.091	0.17	0.093
	18 − 27	29.584	28.71	− 0.03	0.01	0.03	0.113	0.218	0.669	0.782	0.11	− 0.107		
	11 − 17	14.906	14.03	− 0.06	0.01	0.00	0.109	0.180	0.710	0.820	0.11	− 0.105		
	5 – 10	7.755	11.14	0.36	− 0.04	0.47	0.167	0.166	0.667	0.834	0.17	− 0.130		
	< 4.9	3.016	13.67	1.52	− 0.12	1.71	0.527	0.158	0.315	0.842	0.53	− 0.147		
TWI	1.2 − 5.4	34.537	33.04	− 0.04	0.02	− 0.04	0.144	0.237	0.619	0.763	0.14	− 0.121	0.05	0.028
	5.5 − 7.2	37.509	29.72	− 0.23	0.12	− 0.32	0.120	0.248	0.632	0.752	0.12	− 0.110		
	7.3 − 9.4	19.082	24.44	0.25	− 0.07	0.35	0.193	0.191	0.615	0.809	0.19	− 0.138		
	9.5 − 13	7.012	8.32	0.17	− 0.01	0.22	0.179	0.166	0.655	0.834	0.18	− 0.134		
	> 13	1.859	4.48	0.88	− 0.03	0.94	0.364	0.158	0.478	0.842	0.36	− 0.160		
Curvature	Concave (< 0)	31.997	37.57	0.13	− 0.07	0.18	0.381	0.332	0.287	0.668	0.38	− 0.160	0.02	0.010
	Flat (zero)	31.050	39.58	0.12	− 0.07	0.17	0.377	0.343	0.280	0.657	0.38	− 0.160		
	Convex (> 0)	36.953	22.84	− 0.33	0.12	− 0.47	0.242	0.325	0.433	0.675	0.24	− 0.149		
DTS (m)	0 − 140	28.251	68.69	0.89	− 0.83	1.38	0.568	0.221	0.211	0.779	0.57	− 0.139	0.21	0.111
	150 − 320	27.916	12.22	− 0.83	0.20	− 1.37	0.102	0.220	0.678	0.780	0.10	− 0.101		
	330 − 510	22.488	8.75	− 0.95	0.16	− 1.46	0.091	0.204	0.705	0.796	0.09	− 0.095		
	520 − 730	14.939	6.65	− 0.81	0.09	− 1.25	0.104	0.186	0.710	0.814	0.10	− 0.102		
	> 740	6.407	3.69	− 0.55	0.03	− 0.93	0.135	0.169	0.696	0.831	0.13	− 0.117		
SD (Km/Km^2^)	0.1 − 1.2	10.937	10.92	0.00	0.00	− 0.03	0.193	0.178	0.628	0.822	0.19	− 0.138	0.05	0.026
	1.3 − 1.7	23.882	31.02	0.26	− 0.10	0.34	0.252	0.209	0.540	0.791	0.25	− 0.151		
	1.8 − 2.1	28.750	10.77	− 0.98	0.23	− 1.23	0.073	0.223	0.704	0.777	0.07	− 0.083		
	2.2 − 2.6	21.915	32.90	0.41	− 0.15	0.54	0.291	0.204	0.506	0.796	0.29	− 0.156		
	> 2.7	14.516	14.39	− 0.01	0.00	− 0.03	0.192	0.186	0.622	0.814	0.19	− 0.138		
Lithology	Wadi Deposits	5.246	0.00	− 2.89	0.05	− 0.11	0.000	0.062	0.938	0.938	0.00	0.000	0.42	0.229
	Undifferentiated Quaternary Deposits	11.725	17.57	0.41	− 0.07	0.42	0.077	0.066	0.857	0.934	0.08	− 0.086		
	Metasediments	20.018	10.92	− 0.61	0.11	− 0.77	0.028	0.073	0.899	0.927	0.03	− 0.044		
	Intrusive Metagabbro to Metadiorite	6.707	0.00	− 2.89	0.07	− 0.12	0.000	0.063	0.937	0.937	0.00	0.000		
	Older Granite	18.479	0.00	− 2.89	0.21	− 0.26	0.000	0.072	0.928	0.928	0.00	0.000		
	Acid volcanic rocks	7.020	0.00	− 2.89	0.07	− 0.13	0.000	0.063	0.937	0.937	0.00	0.000		
	Gabbroic Rocks	0.780	0.00	− 1.42	0.01	− 0.06	0.000	0.059	0.941	0.941	0.00	0.000		
	Abu’Aggag Fm	4.243	3.62	− 0.16	0.01	− 0.22	0.044	0.061	0.895	0.939	0.04	− 0.060		
	Timsah Fm	3.985	13.02	1.19	− 0.10	1.24	0.169	0.061	0.771	0.939	0.17	− 0.130		
	Ophiolitic Metagabbro	6.483	0.00	− 2.89	0.07	− 0.12	0.000	0.062	0.938	0.938	0.00	0.000		
	Younger Granite	0.087	0.00	− 0.21	0.00	− 0.05	0.000	0.058	0.942	0.942	0.00	0.000		
	Intermediate to Acid Metavolcanic	2.565	7.38	1.06	− 0.05	1.06	0.148	0.060	0.792	0.940	0.15	− 0.123		
	Bahariya FM	3.841	32.97	2.17	− 0.36	2.48	0.443	0.061	0.496	0.939	0.44	− 0.157		
	Basalt	0.006	0.00	− 0.02	0.00	− 0.05	0.000	0.058	0.942	0.942	0.00	0.000		
	Moderate to High Metamorphic Rocks	8.262	14.53	0.57	− 0.07	0.58	0.091	0.064	0.846	0.936	0.09	− 0.095		
	Ring complex	0.553	0.00	− 1.16	0.01	− 0.06	0.000	0.059	0.941	0.941	0.00	0.000		
Radar backscattering	161 – 255	8.110	3.25	− 0.91	0.05	− 1.03	0.090	0.173	0.738	0.827	0.09	− 0.094	0.06	0.033
	132 − 161	22.093	12.80	− 0.55	0.11	− 0.73	0.129	0.204	0.667	0.796	0.13	− 0.115		
	100 − 132	21.953	18.00	− 0.20	0.05	− 0.31	0.183	0.203	0.614	0.797	0.18	− 0.135		
	40 − 100	30.575	45.12	0.39	− 0.24	0.56	0.329	0.229	0.442	0.771	0.33	− 0.159		
	0 – 40	17.269	20.82	0.19	− 0.04	0.17	0.269	0.192	0.539	0.808	0.27	− 0.153		
LD (Km/Km^2^)	0—0.053	17.393	7.37	− 0.86	0.11	− 1.12	0.071	0.192	0.737	0.808	0.07	− 0.081	0.22	0.117
	0.054 − 0.1	28.506	10.77	− 0.98	0.22	− 1.34	0.063	0.222	0.715	0.778	0.06	− 0.076		
	0.11 − 0.16	26.920	11.27	− 0.87	0.19	− 1.21	0.070	0.217	0.713	0.783	0.07	− 0.081		
	0.17 − 0.25	19.028	55.49	1.08	− 0.60	1.53	0.487	0.196	0.317	0.804	0.49	− 0.152		
	0.26 − 1	8.153	15.10	0.62	− 0.08	0.55	0.309	0.173	0.518	0.827	0.31	− 0.158		
LSID (Km/Km^2^)	0 − 0.22	19.826	15.39	− 0.25	0.05	− 0.67	0.152	0.200	0.649	0.800	0.15	− 0.124	0.43	0.234
	0.23 − 0.39	20.276	2.75	− 2.00	0.20	− 2.56	0.026	0.201	0.773	0.799	0.03	− 0.042		
	0.4 − 0.54	20.429	14.31	− 0.36	0.07	− 0.79	0.137	0.201	0.662	0.799	0.14	− 0.118		
	0.55 − 0.72	20.219	0.00	− 3.04	0.23	− 0.59	0.000	0.201	0.799	0.799	0.00	0.000		
	0.73 − 1.2	19.250	67.56	1.26	− 0.91	1.82	0.685	0.198	0.117	0.802	0.69	− 0.112		
Rainfall (mm/year)	6.5 − 8.5	36.192	21.37	− 0.53	0.21	− 0.95	0.166	0.243	0.591	0.757	0.17	− 0.129	0.22	0.120
	8.6 − 10	37.167	63.83	0.54	− 0.55	0.88	0.482	0.246	0.271	0.754	0.48	− 0.153		
	11 − 13	15.054	7.44	− 0.71	0.09	− 1.01	0.139	0.182	0.679	0.818	0.14	− 0.119		
	14 − 16	9.709	7.36	− 0.28	0.03	− 0.52	0.213	0.171	0.616	0.829	0.21	− 0.143		
	17 − 22	1.879	0.00	− 1.28	0.02	− 0.23	0.000	0.158	0.842	0.842	0.00	0.000		

*TWI* Topographic Wetness Index, *DTS* distance to streams, *SD* stream density, *LD* lineament density, *LSID* Lineament Intersection Density; *Bel* belief, *Dis* disbelief, *Unc* uncertainty, *Pls* plausibility.

*Indicate classes where the 0.5 correction factor was applied due to zero wells. W^+^ values for zero-well classes are calculated as ln[(0 + 0.5)/(class area + 0.5)] according to Bonham-Carter^81^.

#### Topographic factors

The topographic characteristics of a region fundamentally control the distribution of hydrological processes, influencing everything from the generation of surface runoff to the location of groundwater recharge. In the Wadi Hodein basin, several key topographic factors were analyzed to assess their impact on groundwater potential, with the statistical weights derived from the WoE and EBF models (Table 2) providing a quantitative measure of each factor’s influence.

**Fig. 6:**
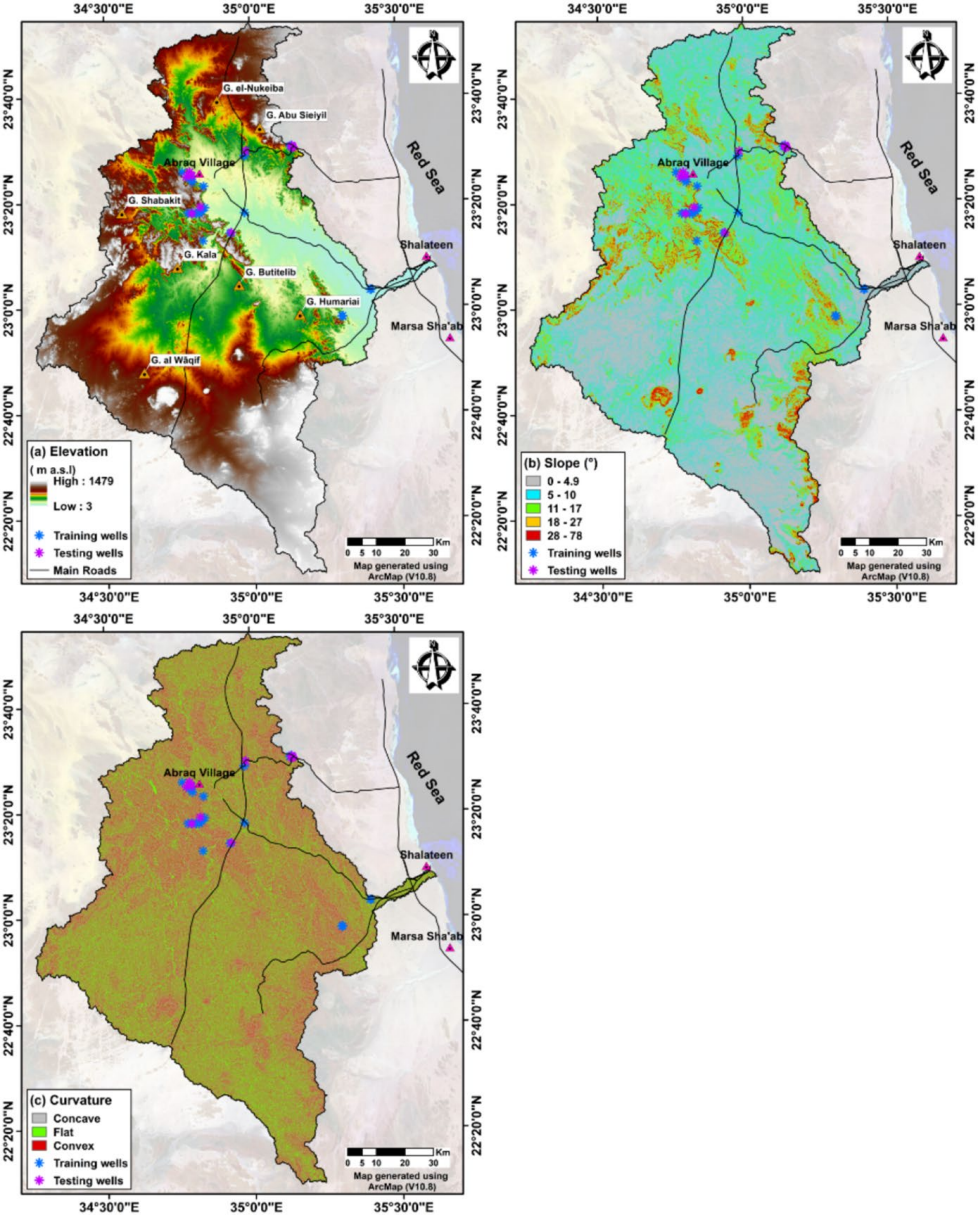
Topographical factors influencing groundwater in Wadi Hodein basin: (**a**) Elevation clipped from ALOS-1 DEM, which is publicly available at the Alaska Satellite Facility Vertex database https://search.asf.alaska.edu/#/, (**b**) Slope angle map, and (**c**) Curvature map. These figures were prepared using ArcGIS Pro v3.6 (https://www.esri.com/en-us/arcgis/products/arcgis-pro/overview).

Elevation is a primary control, dictating climatic conditions and geomorphic processes. As shown in Fig. 6a, the area exhibits a diverse topography, with elevations ranging dramatically from below 3 m to over 1,479 m above sea level. This wide range creates distinct environmental zones. The low-lying areas, often located near main drainage outlets and the Red Sea coast, act as natural collection points for surface runoff generated in the highlands.

Slope gradient directly governs the velocity of surface runoff and, consequently, the potential for water to infiltrate into the ground. The slope map (Fig. 6b) classifies the terrain into various categories. The statistical analysis in Table 2 reveals that gentle slopes (< 4.9°) exert the most significant positive influence on groundwater potential in both models (WoE: 1.71, EBF: 0.527). This is followed by moderate slopes between 5° and 10° (WoE: 1.42, EBF: 0.167). In stark contrast, steeper slopes exceeding 27 exhibit the weakest associations with groundwater occurrence, with negative WoE values and the lowest EBF score (WOE: -0.45, EBF: 0.084).

Curvature describes the shape of the land surface and its influence on water flow convergence or divergence. The terrain was classified into concave (negative values), convex (positive values), and flat (zero-value) zones, as illustrated in Fig. 6c. The findings from the statistical models (Table 2) show that concave areas have a positive influence on groundwater potential (WoE: 0.18, EBF: 0.381), followed closely by flat terrain (WoE: 0.17, EBF: 0.377). Convex slopes exhibit a negligible or slightly negative influence, as seen by their lower WoE and EBF values.

#### Hydrological factors

**Fig. 7:**
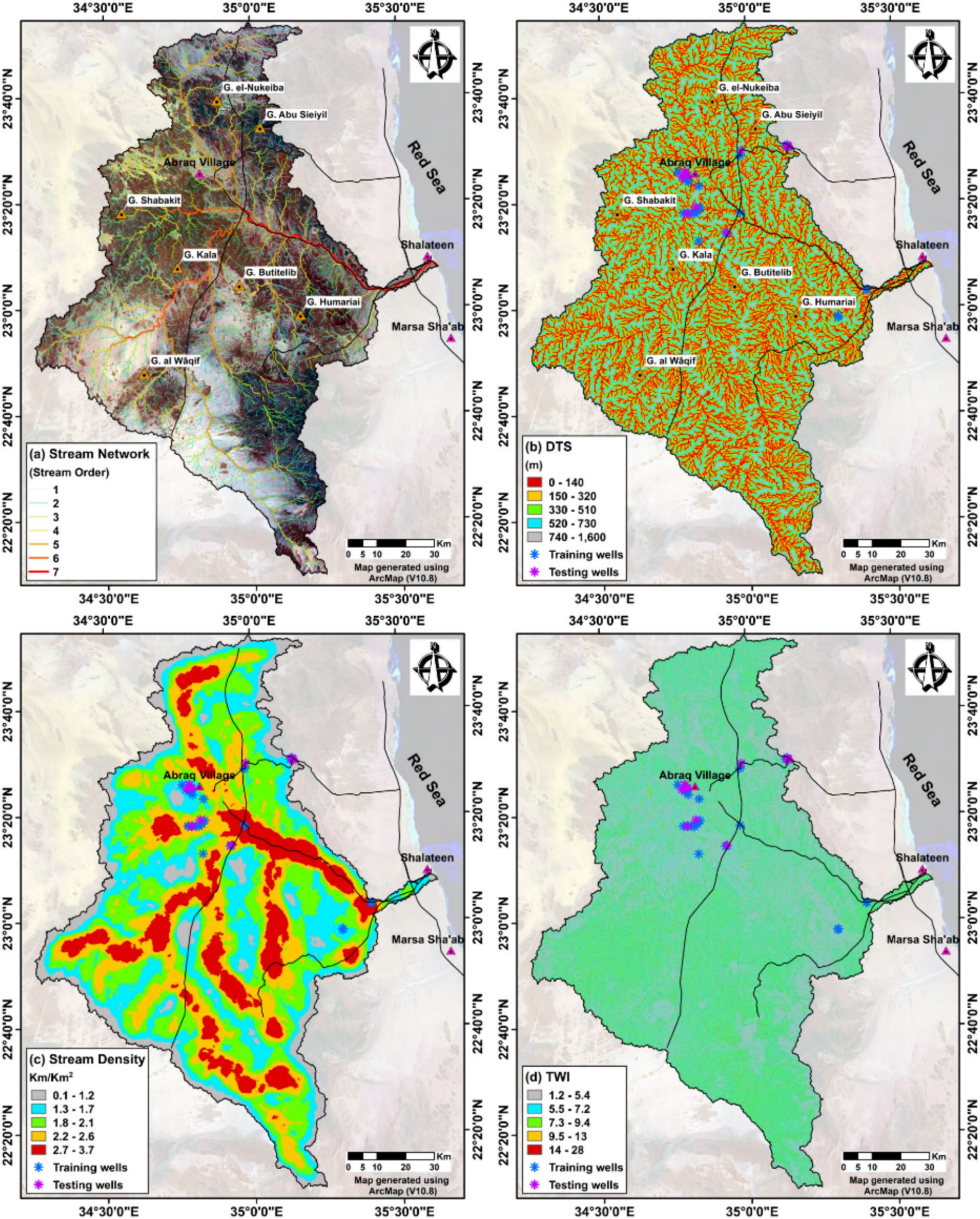
Hydrological factors influencing groundwater in Wadi Hodein basin: (**a**) Stream order map, (**b**) Distance from Stream (DTS) map, (**c**) Stream Density (SD) map, and (**d**) Topographic Wetness Index (TWI) map. These figures were prepared using ArcGIS Pro v3.6 (https://www.esri.com/en-us/arcgis/products/arcgis-pro/overview).

Hydrological site characterization is a critical factor in runoff and recharge processes. Several key factors derived from the drainage network were analyzed, with WoE and EBF weights (Table 2) providing a quantitative measure of their influence on groundwater potential. As shown in Fig. 7a, the study area features a well-developed dendritic stream pattern, particularly near Gebel Shabakit and Gabel Kala in the northwest. This network exhibits high stream orders, with flow directed NW–SE, N-S, and SE-NW toward the Red Sea, defining the primary axes of surface water transport.

Distance to Streams (DTS) dictates proximity to channelized flow. The DTS map (Fig. 7b) shows that areas within 140 m of streams exert the most pronounced positive influence on groundwater potential (WOE: 1.38, EBF: 0.568), emphasizing the role of near-channel infiltration (Table 2). Beyond this distance, influence declines gradually.

Stream Density (SD) measures the efficiency of the drainage network. Analysis of the SD map (Fig. 7c) reveals that moderate-to-high density classes show the strongest correlations with groundwater occurrence (Table 2). The strongest influence is observed in the 2.2–2.6 km/km^2^ class (WOE: 0.54, EBF: 0.291), followed by 1.3–1.7 km/km^2^ (WOE: 0.34, EBF: 0.252).

The Topographic Wetness Index (TWI) identifies areas prone to water accumulation. The strongest positive correlation with groundwater potential is observed in the highest TWI classes (Fig. 7d and Table 2), which represent zones that concentrate surface and subsurface flow^24^. Conversely, the weakest influence was observed in the lowest TWI classes, e.g., ranging from 1.2 to 5.4 (WoE: -0.04, EBF: 0.144) and 5.5 to 7.2 (WoE: -0.32, EBF: 0.120), indicating minimal contribution to groundwater potential.

#### Geological factors

As illustrated in the geological map of the area (Fig. 3), the geological diversity exerts a fundamental control on groundwater occurrence by determining the presence of permeable materials. Statistical weights from the WoE and EBF models (Table 2) quantify the relative influence of each lithological unit on potential groundwater (PGw).

The Bahariya Formation emerges as the most influential unit for groundwater potential. As part of the Nubian Sandstone Aquifer System (NSAS), its thick sandstone sequences provide excellent porosity and permeability. Outcropping significantly in the area (Fig. 3), it shows the strongest positive association with PGw across both models (WoE: 2.48, EBF: 0.443). This aligns with field evidence, as most wells in Wadi Abu Saafa tap this unit, which attains a thickness of 90 m and TDS values ranging from 800 to 2100 ppm. The Timsah Formation, another NSAS component extending westward (Fig. 3), shows a positive but moderate association (WoE: 1.24, EBF: 0.169). In contrast, basement complex rocks exhibit limited influence. Intermediate to Acid Metavolcanic rocks show a weaker association (WoE: 1.06, EBF: 0.148), while Moderate to High Metamorphic Rocks display a modest positive association (WoE: 0.58), with storage restricted to fractures and weathering zones.

**Fig. 8:**
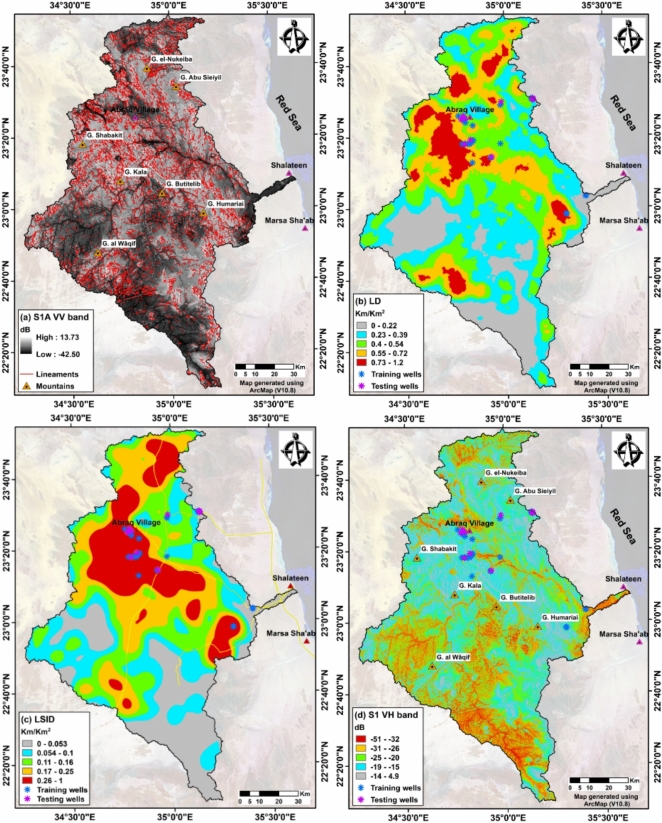
Geological factors influencing groundwater in Wadi Hodein basin: (**a**) Sentinel-1 (S1A) VV polarization which is created and processed by ESA SNAP V9.0 (https://step.esa.int/main/download/snap-download/), overlaid by the resulting automatically extracted lineaments using ArcGIS Pro v3.6 (https://www.esri.com/en-us/arcgis/products/arcgis-pro/overview), (**b**) Lineament Density (LD), (**c**) Lineament Stream Intersection Density (LSID), and (**d**) VH polarizations of S1A classified map. These figures were prepared using ArcGIS Pro v3.6 (https://www.esri.com/en-us/arcgis/products/arcgis-pro/overview).

Beyond exposed units, subsurface features and structural elements play a critical role. Buried channels composed of permeable sand and gravel act as preferential pathways, enhancing recharge rates. Dark-toned areas in Fig. 8a highlight these highly permeable surfaces, supporting their suitability for recharge^95^. Lineament density (LD) also exerts strong control: the 0.17–0.25 km/km^2^ class exhibits the strongest influence (WoE: 1.53, EBF: 0.487), followed by 0.26–1.00 km/km^2^ (WoE: 0.55, EBF: 0.309), reflecting increased permeability in fractured zones (Fig. 8a-b). Notably, in Fig. 8c, the highest lineament intersection density (LSID) class (> 0.73 km/km^2^) shows the strongest association with groundwater occurrence (WoE: 1.82, EBF: 0.685), as fracture intersections enhance permeability and storage^8^. These patterns underscore the role of structural controls in regulating groundwater movement^96^.

Soil texture is a critical geological factor in identifying suitable locations for groundwater recharge processes^24^. The most significant influence was observed in areas with the lowest Rb classes (Fig. 8d), particularly within the ranges of 0–40 (WoE: 0.17, EBF: 0.269) and 40–100 (WoE: 0.56, EBF: 0.329). The low Rb areas in the S1A map (Fig. 8d) are characterized by unconsolidated sediments, such as sand and gravel deposits, which enhance infiltration and facilitate groundwater replenishment^24^.

#### Precipitation

According to the precipitation level (Fig. 2), the WoE and EBF models identify precipitation levels of 8.6–10 mm/year as optimal (WoE: 0.88, EBF: 0.482), while the EBF model also indicates 14–16 mm/year as another favorable range (EBF: 0.213). The concentration of optimal precipitation zones in areas characterized by high lineament density (LD: 0.17–0.25 km/km^2^) and high lineament intersection density (LSID: > 0.73 km/km^2^) is notable.

### Groundwater potential zone maps

**Fig. 9:**
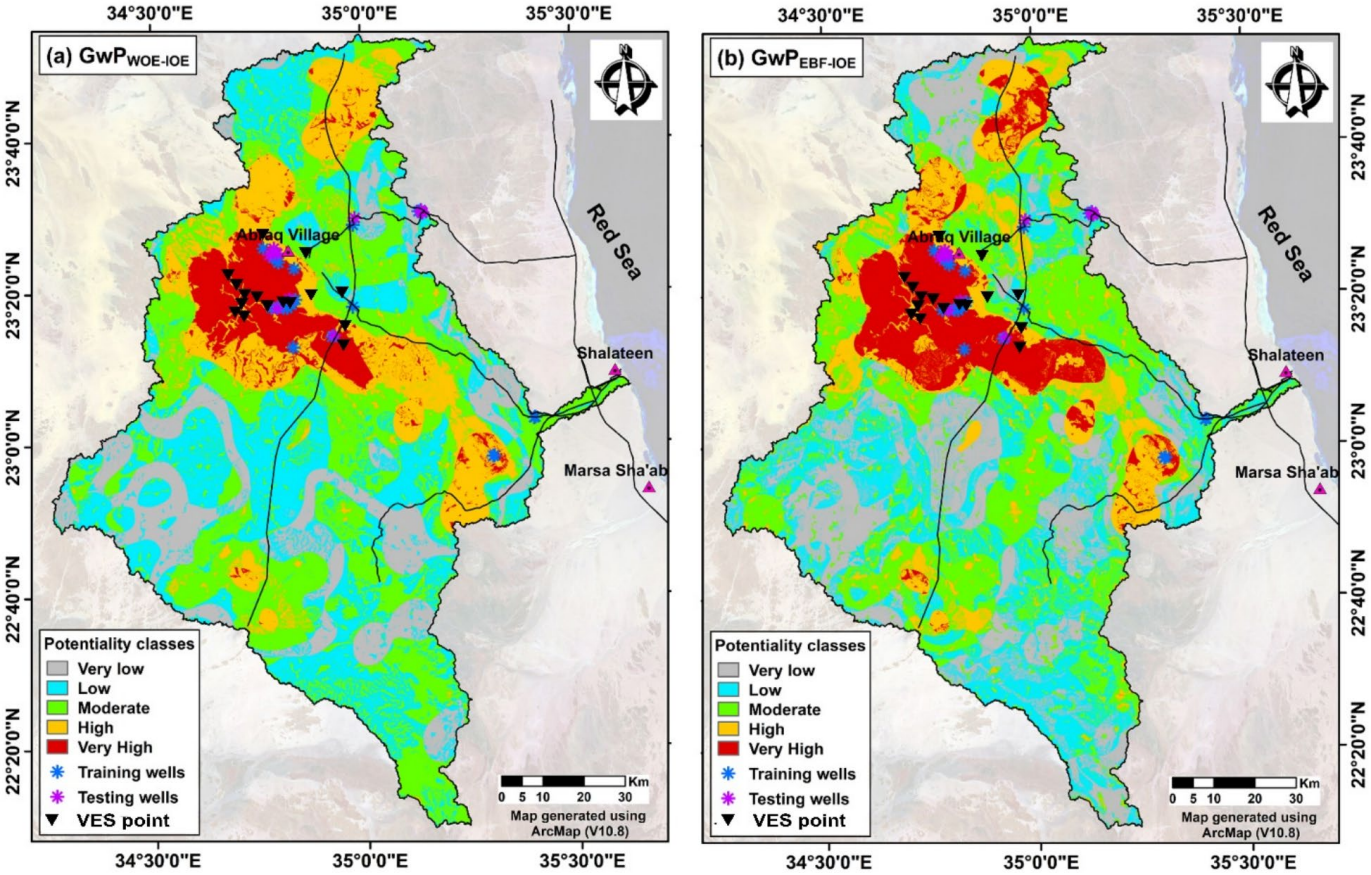
Groundwater Potentiality (GwP) maps of Wadi Hodein basin overlaid by productive testing groundwater wells: (**a**) Weight of Evidence (GwP_WOE-IOE_) and (**b**) Evidential Belief Function (GwP_EBF-IOE_). These figures were prepared using ArcGIS Pro v3.6 (https://www.esri.com/en-us/arcgis/products/arcgis-pro/overview).

Using the statistical methodologies outlined earlier, two groundwater potential (GwP) maps were generated through hybrid models that integrate the Index of Entropy (IOE) with the Evidential Belief Function (EBF) and Weight of Evidence (WOE) models (Fig. 9). This ensemble-based bivariate decision framework enables a comprehensive evaluation of groundwater potential.

Discrepancies between the resulting maps can be attributed to the factor rankings analyzed in Table 2. The IOE model was employed to address imbalances among the ten influencing factors by assigning predictive weights, thereby ensuring an objective assessment of their relative importance. According to the IOE model’s weight distribution, the lineament stream intersection density (LSID) exhibited the highest value (0.234), followed closely by lithology (0.229), rainfall (0.120), lineament density (LD) (0.117), distance to stream (DTS) (0.111), slope (0.093), radar intensity (0.033), topographic wetness index (TWI) (0.028), and stream density (SD) (0.026), with curvature displaying the lowest weight (0.01).

Table 3 presents a quantitative comparison of the spatial extent of each GwP zone as delineated by the EBF-IOE and WOE-IOE models. The GwP_WOE-IOE_ model classifies approximately 7.5%, 16.87%, 33.57%, 27.76%, and 14.3% of the area into very high, high, moderate, low, and very low groundwater potential zones, respectively (Table 3, Fig. 9a). In contrast, the GwP_EBF-IOE_ model identifies 11.75%, 15.79%, 28.98%, 23.2%, and 20.32% of the area within the same classes. The GwPEBF-IOE map (Fig. 9b) indicates that high groundwater potential zones are primarily concentrated in the northwestern region, particularly within the Nubian Sandstone area, extending between Abraq village and the Shabakit, Kala, and Butitelib mountains. This zone also extends eastward along Wadi Hodein and encompasses the fractured basement rocks in the central part of the region. This spatial distribution is largely influenced by critical factors such as LSID, lithology, LD, DTS, and precipitation. The presence of a highly fractured zone suggests that subsurface fractures may act as conduits for groundwater movement and accumulation within the bedrock terrain.

**Table 3:** Proportion of groundwater (Gw) inventory points distributed across the GwP classes.

Models	Zones	Area (km^2^)	Area (%)	Gw points (%)
GwP_WOE-IOE_	Very low	1640.92	14.30	2.50
	Low	3186.68	27.76	12.50
	Moderate	3852.60	33.57	12.50
	High	1936.77	16.87	12.50
	Very High	860.74	7.50	60.00
GwP_EBF-IOE_	Very low	2332.44	20.32	0.00
	Low	2662.35	23.20	15.00
	Moderate	3321.78	28.94	7.50
	High	1812.80	15.79	12.50
	Very High	1348.35	11.75	65.00

A GIS-based overlay analysis further indicates that 67.5% of the recorded potential groundwater (PGw) inventory aligns with the high- and very-high-potential zones identified in the GwPEBF-IOE model (Table 3). Notably, no PGw points were found within the very low potentiality class of the GwPEBF-IOE map, unlike the GwPWOE-IOE model. Although the WOE model has limitations in quantifying uncertainty within factor subclasses, it remains valuable in illustrating the influence of individual subcategories across the area.

These findings underscore the effectiveness of the hybrid ensemble approach combining the IOE and EBF models. This methodology accounts for the complex interactions among geographical, geological, structural, climatic, and hydrological factors while minimizing subjectivity and uncertainty^33^. The results align with previous studies by Pourghasemi and Beheshtirad^88^, Althuwaynee et al.^84^, Tahmassebipoor et al.^94^, and Baduna Koçyiğit and Akay^97^, which highlight the advantages of Dempster-Shafer theory in predictive mapping by incorporating uncertainty modeling alongside favorable zone delineation.

Accordingly, this study demonstrates the cost-effective integration of open-source SAR remote sensing datasets (S1A, ALOS-DEM, and IMERG) with ancillary geospatial data, using hybrid IOE with EBF and WOE modeling for GwP mapping in the Wadi Hodein basin.

### Sensitivity analysis for predictor dependence

To assess whether retaining correlated predictors artificially inflated predictive performance or biased the final groundwater potential map, a sensitivity analysis was conducted. Three alternative WOE models were generated by systematically removing one factor from each strongly correlated pair. Model B (LSID removed), Model C (SD removed), and Model D (TWI removed), all compared against the original Model A (all ten factors retained).

Spatial correlation between the original and alternative models exceeded r = 0.89 in all cases, with changes in high-potential zone classification of less than 8%. The most noticeable change occurred when LSID was removed (Model B), with the high-potential area decreasing by 7.8% (r = 0.91), suggesting that lineament intersection density provides unique information about fracture connectivity not fully captured by LD alone. Model C (SD removed) showed a 3.2% decrease (r = 0.94), while Model D (TWI removed) showed a 4.5% decrease (r = 0.89).

These results demonstrate that while predictor dependence exists, the overall groundwater potential pattern is robust. The structural group (LD and LSID) shows the highest sensitivity, which is expected given that fault-controlled recharge is the dominant process in the structurally controlled aquifers (SCAq).

### Validation of groundwater potentiality models

**Fig. 10:**
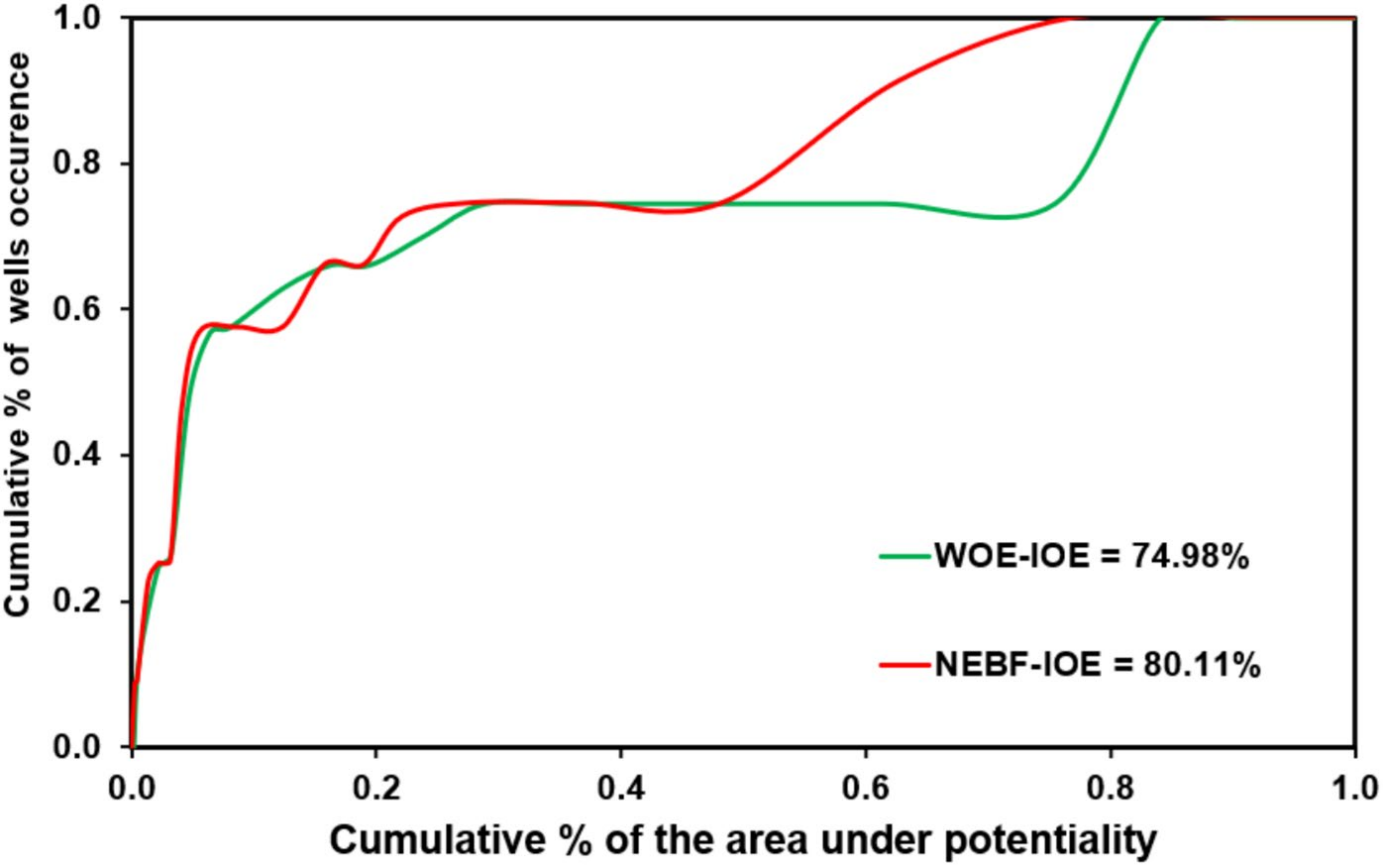
Area under the curve of receiver operating characteristic (AUC-ROC) plot for predictive models of potentiality maps. This figure was prepared using Microsoft Excel 365V 2606 (https://www.microsoft.com/en/microsoft-365/Excel).

To provide a robust assessment of model performance and uncertainty, a repeated fivefold CV was conducted with 5 iterations, as described in Section "Validation of groundwater potential (GwP) models". This approach generated a distribution of AUC values for each model, enabling the calculation of mean performance and associated uncertainty (standard deviation). Figure 10 presents the distribution of AUC values from the 5 iterations of fivefold CV for both the WOE-IOE and EBF-IOE models. The EBF-IOE model achieved a mean AUC of 0.801 (SD =  ± 0.034), with AUC values ranging from 0.732 to 0.858. The WOE-IOE model achieved a mean AUC of 0.749 (SD =  ± 0.041), with values ranging from 0.708 to 0.821. The consistently higher mean AUC and narrower confidence interval of the EBF-IOE model indicate superior predictive performance and greater stability compared to the WOE-IOE model. The relatively narrow confidence intervals (width < 0.024 for both models) indicate that the performance estimates are reliable and not overly sensitive to the specific random partition of the training and testing data (Table 4).

**Table 4:** Repeated fivefold cross-validation results (5 iterations) for WOE-IOE and EBF-IOE models.

Model	Mean AUC	Standard deviation ( ±)	95% confidence interval	Min AUC	Max AUC
WOE-IOE	0.749	0.041	0.737 – 0.761	0.708	0.821
EBF-IOE	0.801	0.034	0.791 – 0.811	0.732	0.858

The WOE-IOE model exhibited strong predictive capability, attaining an AUC of 0.7498 (74.98%), indicating a good fit for the testing dataset. In contrast, the EBF-IOE model demonstrated superior performance, achieving an AUC of 0.8011 (80.11%), which meets the threshold for very good performance in environmental sciences. Remarkably, the EBF-IOE model outperformed the WOE-IOE approach, yielding a 5.13% improvement, underscoring the efficacy of EBF ranks in capturing complex non-linear dependencies between groundwater occurrence and environmental variables, and thus reducing uncertainty^97^. This effectiveness is particularly evident in complex geological settings, as demonstrated by Pourghasemi and Beheshtira^88^, who achieved a success rate of 89% using an individual EBF model for GwP mapping in hard-rock geologic terrain of southwestern Nigeria. These results suggest the model’s applicability to sparse groundwater datasets in Wadi Hodein, a critical attribute for effective groundwater potential mapping in structurally controlled aquifers, and align with prior studies on probabilistic modeling in hydrogeology^96,98^.

Comparative validation confirms that the EBF-IOE hybrid model (AUC = 0.801) outperforms the WOE-IOE model (AUC = 0.749), with the improvement attributable to the integration of Dempster–Shafer theory within the EBF framework, which enhances uncertainty handling under heterogeneous geological conditions. Spatial agreement between observed groundwater occurrences and predicted potential zones was assessed using frequency ratio analysis. The EBF-IOE model assigned 65% of observed PGw points to the “very high” potential class and 12.5% to the “high” class, resulting in 77.5% of observations concentrated within the top two categories, which together occupy only 27.5% of the study area. In comparison, the WOE-IOE model allocated 60% and 12.5% of PGw points to the “very high” and “high” classes, respectively (72.5% total), covering 24.4% of the area. These results confirm the strong predictive capability of both models, while emphasizing the improved performance of the hybrid approach in delineating priority exploration zones. The findings also highlight the effectiveness of integrating ensemble-based probabilistic modeling with open-access SAR data for cost-efficient GwP assessment.

Model validation was further supported using 17 independent VES points that were excluded from the training phase. Of these, 14 points located within high-potential zones exhibited favorable aquifer characteristics, including appropriate thickness and resistivity values indicative of good groundwater quality (Table S3). The remaining three points, situated in low-potential zones, were characterized by high resistivity and limited aquifer thickness, thereby confirming the model’s capability to accurately identify low-yield or non-productive conditions. Supplementary Figure S1 presents representative resistivity curves from high-potential zones, illustrating variations in lithological structure, saturation levels, and layer thickness.

A comprehensive evaluation of all validation metrics confirms the robustness of the EBF–IOE hybrid framework for groundwater potential mapping within the structurally controlled Wadi Hodein aquifer. The model yielded a mean repeated cross-validation AUC of 0.801 (± 0.034) and a spatially blocked cross-validation AUC of 0.764 (± 0.052), demonstrating consistent predictive performance under both random and spatially independent validation schemes. In addition, it successfully delineated 77.5% of the observed groundwater potential (PGw) locations within high and very high potential zones. Independent validation using vertical electrical sounding (VES) data further corroborated the model’s reliability, with all predicted high-potential sites (14/14) confirmed by geophysical evidence. Overall, these findings indicate that the EBF–IOE model exhibits strong predictive accuracy, well-controlled uncertainty, and reliable spatial transferability, rendering it highly suitable for groundwater exploration in structurally complex and data-scarce aquifer systems (Table 5).

**Table 5:** Summary of validation metrics for EBF-IOE and WOE-IOE models.

Validation metric	WOE-IOE	EBF-IOE
Repeated CV Mean AUC (5 iterations)	0.749 (± 0.041)	0.801 (± 0.034)
PGw points in High + Very High Zones (%)	72.5%	77.5%
Independent VES Validation Accuracy*	12/14 (85.7%) *	14/14 (100%) *

*Note: VES points evaluated within the respective high-potential zones of each model.

### Subsurface controls on aquifer heterogeneity from geophysical methods

While the EBF-IOE model successfully identifies high-potential zones at a regional scale, geophysical investigations reveal complex subsurface structures that govern significant local-scale variability in well yield and water quality within these zones.

**Fig. 11:**
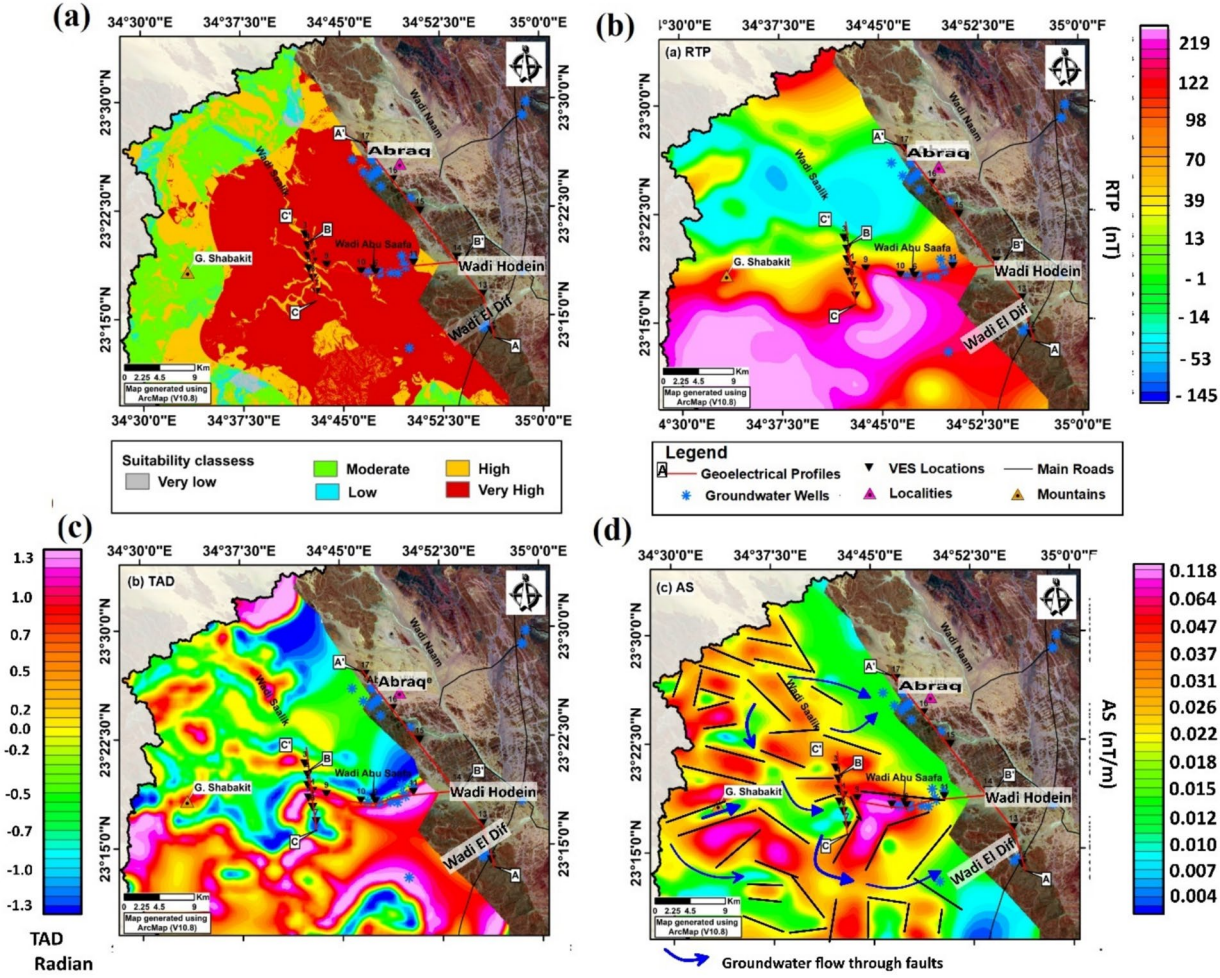
Aeromagnetic data analysis: (**a**) Clipped GwP_EBF-IOE_ using the same boundary as the aeromagnetic survey, (**b**) Reduction-to-the-pole (RTP) aeromagnetic map over the western part of Wadi Hodein basin, (**c**) the Tilt Angle Derivative (TAD) map, and (**d**) Analytical Signal (AS) map. These figures were processed by Oasis Montaj v. 8.3.3. (https://www.seequent.com/products-solutions/oasis-montaj/), and all maps were prepared using ArcGIS Pro v3.6 (https://www.esri.com/en-us/arcgis/products/arcgis-pro/overview).

The Reduced-to-Pole (RTP) magnetic map (Fig. 11b) highlights dominant structural trends, particularly in the northwestern region. This area displays subdued magnetic amplitudes in contrast to the strong magnetic signatures of exposed basement rocks to the south. Sharp east–west trending boundaries suggest fault systems within the basement, which are critical for groundwater movement and storage. This is corroborated by the occurrence of natural springs and shallow wells along these structural contacts. Notably, the model’s high groundwater potential zones align closely with these features.

Analysis of the tilt angle derivative (TAD) map (Fig. 11c) reveals that the northern part of the Wadi Hodein basin is dominated by NW–SE trending linear anomalies, interpreted as alternating graben and horst structures resulting from normal faulting in the basement. These structural complexities are consistent with the dominant trends observed in the drainage stream order and stream density maps (Fig. 7a,c). Additionally, the presence of a shallowly buried channel, identified in the Sentinel-1 (S1A) VV polarization map (Fig. 8a), further supports this correlation.

The analytic signal (AS) map (Fig. 11d) reveals a significant spatial correlation between high AS amplitudes and shallow basement uplifts. In contrast, areas with low relief demonstrate greater recharge potential, as these depressions allow for more effective water infiltration. The intermediate AS amplitudes observed in the Nubian Sandstone suggest a hydrostratigraphic relevance that may facilitate localized recharge, particularly in regions such as the upstream of W. Abu Saafa (E-W trend) and the eastern Abraq area (Fig. 11d). Most of the delineated magnetic lineaments exhibit trends of NW–SE in the northern region, E-W around W. Abu Saafa, and N-S in the southern area.

Structural isolation of certain sub-basins, particularly in the downstream regions of W. Hodein and W. Abu Saafa, is a key finding (Fig. 11d). This isolation is caused by N-S basement uplifts, identified by high AS anomalies, resulting in highly variable groundwater potential across the area (Fig. 11a). This structural contact between the uplifted basement and the Nubian Sandstone aquifer, often defined by normal faults, is a preferential flow path and the primary location for springs and successful wells.

To further delineate the hydrostratigraphic framework, three geoelectrical sections were constructed across key geological contacts between basement rocks and the Nubian Sandstone, informed by well-drilling data (Table 1), surface topography, and geological exposures (Fig. 2).

**Fig. 12:**
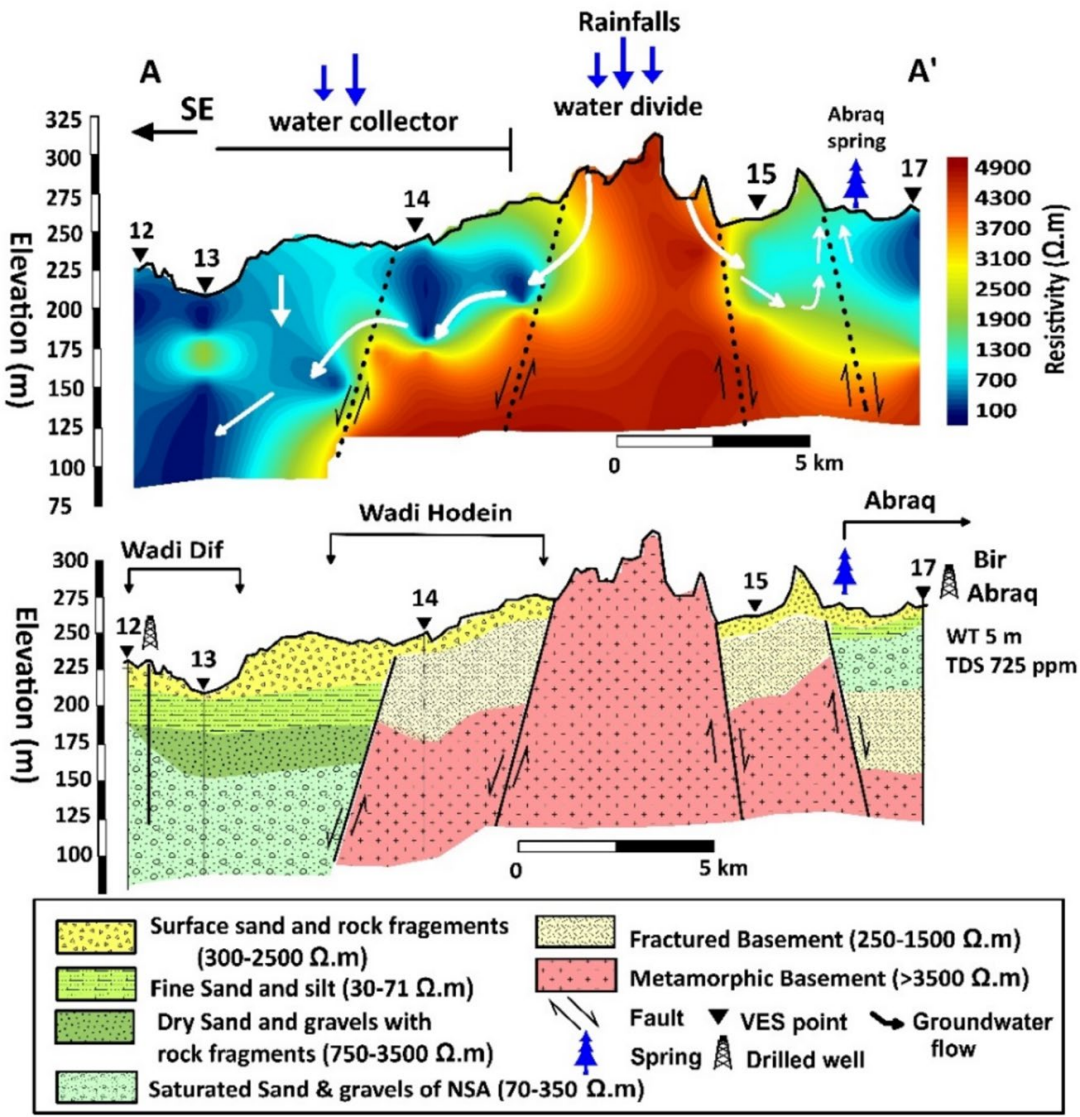
Geoelectrical Cross-Section AA.’ The section runs NW–SE (for location see Fig. 8b), crossing W. Hodein. (**a**) The resistivity distribution reveals lateral variations caused by faults and basement uplifts. (**b**) Geological interpretation of the cross-section, constrained by the drilled borehole along the profile.

The geoelectrical profile A-A' (Fig. 12) extends from the W. El-Dif well in the southeast to the Abraq spring in the northwest, crossing the W. Hodein mainstream. The profile reveals variations in basement bedrock depth and in the characteristics of the Nubian Sandstone (Timsah and Bahariya formations), influenced by subsurface faults. Metasediment exposures near W. Hodein act as a barrier, dividing the area into two distinct hydrological systems. South of W. Hodein, near the W. El-Dif watershed, thick deposits of saturated sand and gravel from the Bahariya Formation (NSAS) exhibit resistivity values from 78 to 350 Ω·m and thicknesses over 100 m. In contrast, the Abraq area features a shallow aquifer zone within the fractured basement and Nubian Sandstone, evidenced by shallow dug wells (10 m depth) and springs.

**Fig. 13:**
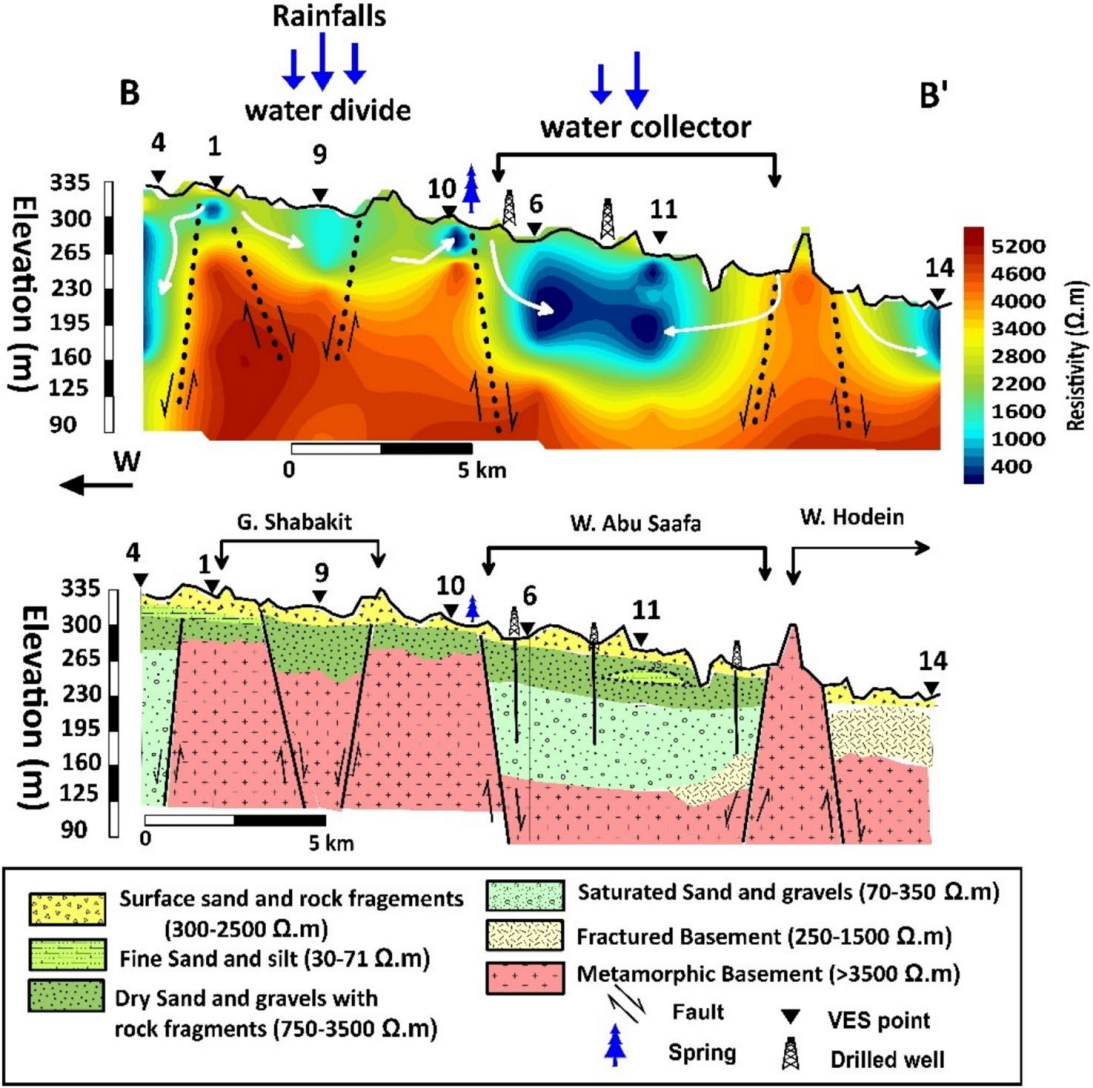
Geoelectrical Cross-Section BB’: The section runs E-W (for location see Fig. 8b), along W. Hodein and W. Abu Saafa. (**a**) The resistivity distribution reveals lateral variations caused by faults and basement barriers. (**b**) Geological interpretation of the cross-section, constrained by the drilled borehole along the profile.

Profile B-B' (Fig. 13), an east–west geoelectrical transect, reveals the Nubian Sandstone aquifer’s architecture and structural controls from W. Hodein to W. Abu Saafa. Integrated resistivity, magnetic, and well data show a heterogeneous aquifer partitioned into three compartments by basement topography and faults. The eastern compartment near W. Hodein consists of fractured basement and Quaternary deposits with low groundwater potential. In contrast, the central W. Abu Saafa compartment is a productive graben bounded by basement uplifts, filled with thick Nubian Sandstone (Bahariya Formation), with resistivities of 17.5–111 Ω·m corresponding to TDS of 824–2120 ppm. The western compartment of W. Abu Saafa is generally an uplifted block with high resistivity (2100–3000 Ω·m), indicating low potential. However, localized recharge occurs at VES 10 (83 Ω·m, ~ 10 m thick) close to Abu Saafa Spring.

**Fig. 14:**
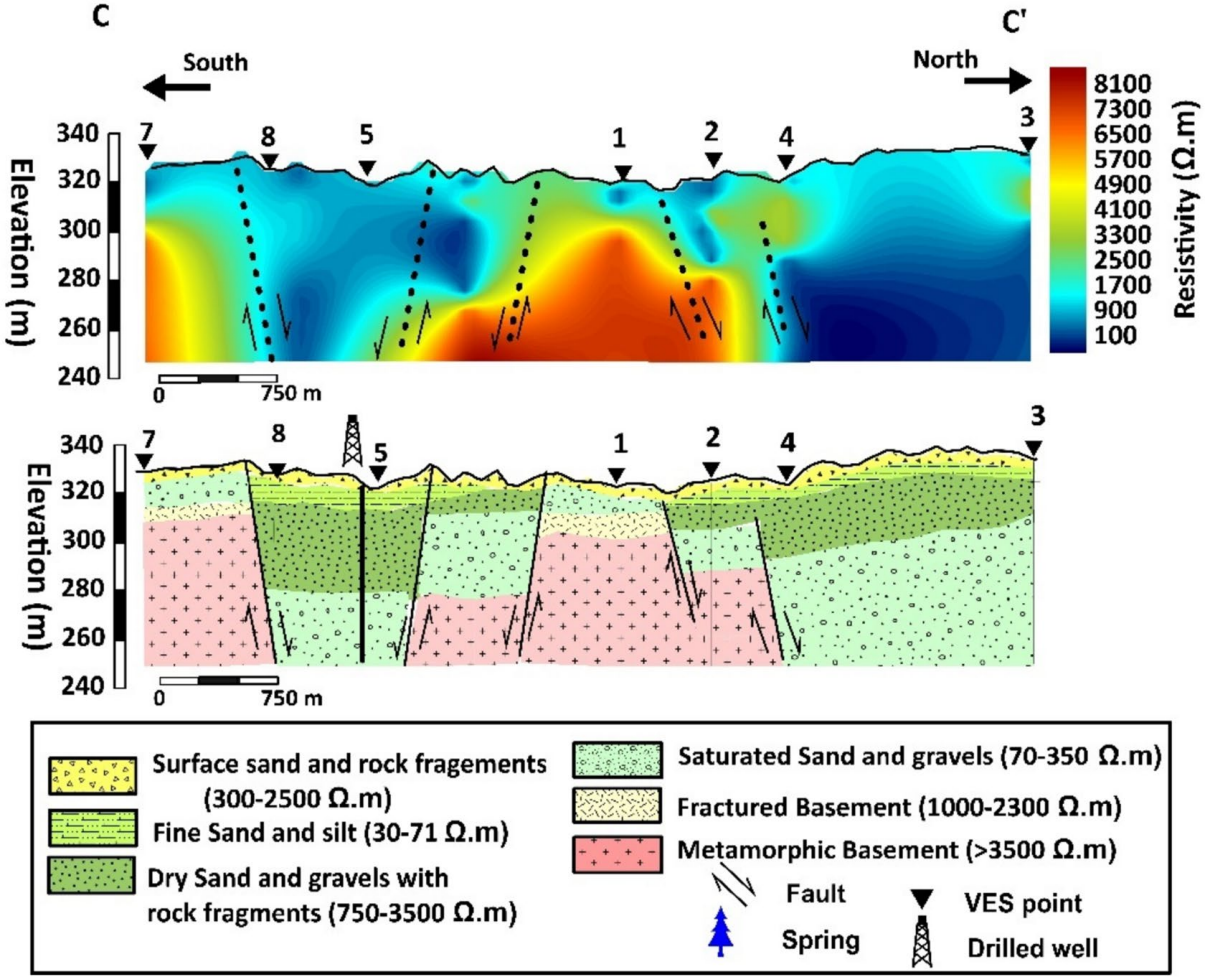
Geoelectrical Cross-Section CC’: The section runs N-S (for location see Fig. 8b), crossing the streamlines of W. Abu Saafa. (**a**) The resistivity distribution reveals lateral variations caused by faults and basement uplifts. (**b**) Geological interpretation of the cross-section, constrained by the test borehole close to VES 5.

The cross-section C–C' (Fig. 14) characterizes the aquifer in the inlets of W. Abu Saafa on the western side. The resistivity distributions along this profile suggest a promising aquifer zone, with resistivity values ranging from 119 to 391 Ω·m. The thickness of the aquifer varies between 70 and 150 m due to the influence of the faulting system. The groundwater depth along the profile ranges from 25 to 65 m below the surface. Notably, the area between VESs 3 and 4 on the northern side, and the region between VESs 5 and 8, demonstrate good groundwater potential for drilling. The latter is identified as a graben in the basement surface, as depicted in the geoelectrical cross-section (Fig. 14) and the TAD magnetic map (Fig. 11b).

**Fig. 15:**
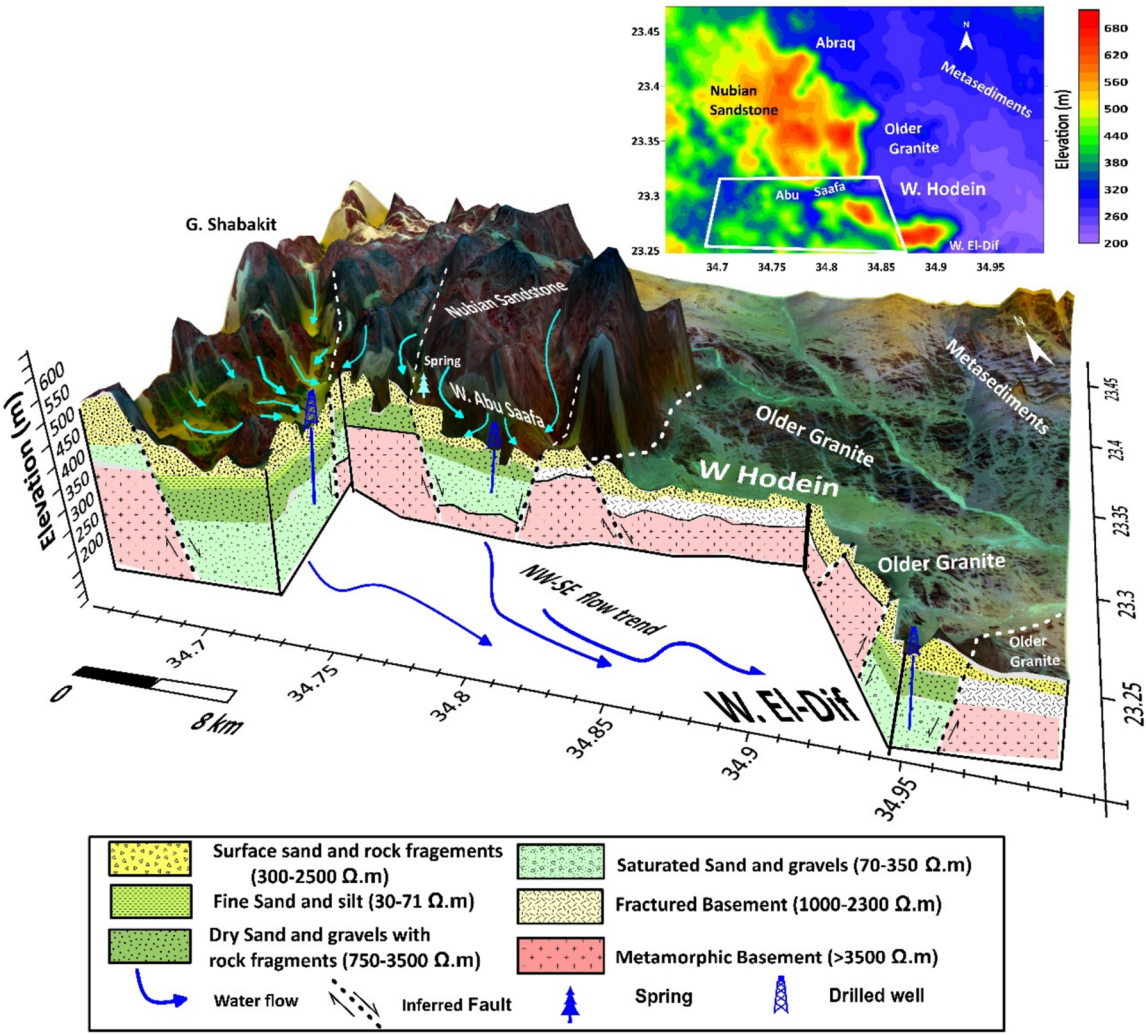
A 3D block diagram depicting the site conceptual flow model derived from geological and hydrogeophysical findings, indicating the connectivity of NSAS through NW–SE and E-W trends. The upper inset shows a high-resolution DEM of the modeled region, highlighted by a blank area (indicated by a white rectangle). Surface geological units are identified from the geological map (Fig. 3), while subsurface layers are obtained from DC resistivity findings and existing borehole records. Common fault trends are inferred from magnetic and remote sensing datasets. These figures were prepared using ArcGIS Pro v3.6 (https://www.esri.com/en-us/arcgis/products/arcgis-pro/overview) and Surfer v31 (https://www.goldensoftware.com/products/surfer/trial/).

The 3D site conceptual flow block (Fig. 15), constructed from geophysical and topographical data, elucidates the complex interplay of subsurface structures in regulating groundwater dynamics within the NSAS and Precambrian basement terrains. This block highlights key groundwater potential zones, notably in Wadi Abu Saafa and the western tributaries, where geological features such as grabens facilitate the accumulation and lateral movement of groundwater across various fault orientations, including NW–SE, E-W, and NE-SW trends. The analysis reveals the promising potential of the grabens in Wadi Abu Saafa and the western streams in conjunction with the eastern part of Wadi El-Dif. These findings are corroborated by the geological map (Fig. 2), the Sentinel-1 (S1A) VV map (Fig. 8a), and the AS magnetic map (Fig. 11d).

The geoelectrical profiles provide quantitative evidence linking structural setting to GwP class. VES points located within magnetic-defined basement grabens (central W. Abu Saafa) exhibited significantly lower resistivity (17–111 Ω·m) and greater aquifer thickness (90–100 m), corresponding to the very high GwP class. In contrast, points on uplifted basement blocks (western compartment) showed mean resistivity exceeding 2100 Ω·m and thickness below 15 m, falling within the low GwP class. The fault-bounded basin south and west of W. Hodein represents the high GwP class, with an average resistivity of 250 Ω·m and an average thickness of 110 m. The metasediment barrier, classified as very low potential, exhibited the highest resistivity (> 3000 Ω·m) and thinnest aquifer thickness (10–20 m).

This integration of magnetic and VES data refines the WOE-based potential mapping by explicitly quantifying how basement relief and fault networks control groundwater accumulation—information that surface-based remote sensing alone cannot provide.

## Discussion

The results demonstrate the effectiveness of integrating IOE with bivariate statistical models (EBF and WOE) for groundwater potential mapping in the structurally complex Wadi Hodein basin. The geospatial factor analysis highlights the dominant control of structural features, with LSID and lithology receiving the highest predictive weights from the IOE model. This quantitative evidence confirms that zones of high fracture density and lithological contacts are the primary determinants of groundwater occurrence in this hard-rock terrain, consistent with previous studies^8,96,98^. The strong performance of LSID underscores a key advancement over previous regional assessments^52^, which underestimated structural lineaments.

While the initial well inventory was limited and presence-biased, integrating dry wells as absence points and rigorously validating the results using VES data (including low-potential sites) strengthens the reliability of the groundwater potential maps (Table S3). The agreement between the statistical models and the geophysical interpretation of bedrock structures confirms the robustness of the applied methodology.

A key finding is the critical disconnect between regional-scale potential mapping and local-scale aquifer heterogeneity, a gap bridged through detailed geophysical investigation. Incorporating geophysical methods significantly reduces ambiguity inherent in GIS-based models^18^ by validating groundwater occurrence, reducing uncertainty, and minimizing drilling risks. These techniques bridge the gap between regional analysis and precise well siting by directly exploring subsurface structures.

While the GIS-based EBF-IOE model successfully identified the northwestern region as a high-potential area (Fig. 9b), geophysical data revealed this zone to be a structurally complex and compartmentalized system. Aeromagnetic data (TAD and AS maps) and geoelectrical profiles consistently image a basement surface dissected by normal faults, resulting in a mosaic of grabens and horsts. This structural complexity directly influences hydrogeological dynamics, as evidenced by the wide variation in well depths—from shallow springs in Abraq to depths exceeding 100 m in Wadi Abu Saafa^98^—and diverse recharge sources associated with these faults. Specifically, the variability in well depths and TDS within this high-potential zone is closely correlated with the intersection of NW–SE and N-S trending faults.

This underlying structural architecture has significant implications for water resource development. As illustrated in Profile B-B' (Fig. 13), the central graben of Wadi Abu Saafa forms a productive sedimentary basin (Bahariya Formation), whereas adjacent horsts function as hydrological barriers. This structural control explains the sharp contrast in well yields and aquifer thickness observed over short distances, a variability that surface data alone cannot predict. Furthermore, the integration of magnetic and resistivity data demonstrates that these fault systems also govern recharge pathways. A clear spatial correlation exists between the NW–SE faults, surface drainage patterns (Fig. 7a), buried channels (Fig. 8a), and spring locations, confirming that these structures act as preferential conduits for infiltration, facilitating recharge to both sandstone and fractured basement aquifers.

The 3D conceptual flow block (Fig. 15) synthesizes all datasets, illustrating that the NSAS in Wadi Hodein comprises fault-bounded blocks. Groundwater accumulates in deep sediment-filled grabens (e.g., W. Abu Saafa), while basement horsts force lateral flow along faults or discharge as springs. The grabens in Wadi Abu Saafa, western streams, and western Wadi El-Dif offer favorable conditions for recharge and accessibility, corroborated by the geological map (Fig. 3), Sentinel-1 VV map (Fig. 8a), and AS magnetic map (Fig. 11d).

These findings have direct implications. First, drilling campaigns must target specific structural targets (sediment-filled grabens, major fault intersections) within broad high-potential zones, guided by geophysical data. Second, the compartmentalized aquifer requires a multi-cell management strategy, with each compartment managed based on its own recharge rate and storage capacity. Finally, this study demonstrates a replicable methodology integrating open-source satellite data with targeted geophysical surveys for data-sparse, arid basement terrains. Ignoring this multidisciplinary approach impedes accurate groundwater delineation and effective resource management.

However, some limitations must be considered. First, GIS-based models rely on input data quality and resolution. IMERG precipitation data may not capture localized rainfall events in arid environments. In other global places, the lineament and fault maps derived from this DEM and satellite imagery are also subject to interpreter bias and are limited to surface expressions depending on the local geological setting, which may not always extend to or accurately represent subsurface structures at aquifer depth. Second, statistical models have inherent uncertainties. WOE assumes conditional independence among themes, rarely satisfied in complex natural systems. While IOE mitigates this, residual uncertainty remains. Model accuracy is also limited by the spatial distribution of training data (PGw inventory points). Third, the geophysical methods, while critical for validating and refining the GIS-based predictions, also have inherent constraints. Aeromagnetic data cannot directly detect groundwater, but it is valuable for identifying subsurface faults that influence groundwater flow by highlighting magnetic variations between rock types. Its interpretation is non-unique; different geological configurations can produce similar magnetic signatures. Furthermore, the spatial coverage of VES points, though the main streams in the obtained high potential zone, remains sparse relative to the area’s total geological complexity, leaving potential for undiscovered heterogeneities between survey lines. Nevertheless, geophysical studies within the delineated high-potential zones remain limited, with existing research primarily focusing on wadi deltas along the shoreline and utilizing vertical electrical sounding (VES) surveys (e.g.^99,100^).

Despite the acknowledged limitations of predictor dependence in WOE modeling, the strong spatial correlation between models with and without correlated factors (r > 0.89) and the independent geophysical validation using 17 VES points confirm that the resulting groundwater potential map reliably delineates favorable zones in this structurally controlled aquifer. The weights should be interpreted as descriptive measures of spatial association, and the final map as a relative index rather than absolute probabilities.

Finally, although the study successfully identified structural compartmentalization of the aquifer, it did not incorporate direct hydrogeological time-series data, such as seasonal water-table fluctuations, stable isotopes, or groundwater ages. Such data would be invaluable for quantifying actual recharge rates, confirming groundwater flow directions and velocities inferred from the structural model, and understanding the connectivity between different buried structures. The current exploratory framework provides a robust static framework of where groundwater is likely to occur. Still, the inclusion of dynamic hydrological data in future research is essential to fully understand the groundwater sustainability challenges.

To address the inherent uncertainties and constraints identified in this study, a multi-tiered program of supplementary investigations is recommended. These future research directions aim to refine the conceptual model, quantify the groundwater budget, and enhance the precision of exploration targeting within the Wadi Hodein basin. A systematic sampling campaign across all identified aquifer compartments (e.g., W. El-Dif, central graben of W. Abu Saafa, Abraq spring) for stable isotopes and radioactive age tracers is critical. In addition, the current conceptual model (Fig. 15) should be translated into a transient numerical model (e.g., using MODFLOW or FEFLOW).

## Conclusion

This study underscores the critical importance of an integrative, multi-method approach for accurate groundwater potential mapping in the structurally complex arid region of the Wadi Hodein basin. By combining geophysical data with traditional GIS-based statistical modeling, this research shifts from regional probabilistic predictions to a detailed, process-oriented understanding of the hydrogeological system.

The primary finding of this research establishes structural controls (NNW-SSE, E-W, and N-S faults and basement topography) as the principal drivers of groundwater dynamics. While ensemble statistical models (EBF-IOE and WOE-IOE) successfully delineated regional high-potential zones, their inability to account for significant local variations in well yield and water quality reveals a critical limitation. This underscores a key hydrogeological paradigm in arid regions: surface indicators of groundwater potential often mask a subsurface reality that is fundamentally discontinuous and compartmentalized.

The integration of geophysical methods was indispensable for resolving this complexity. Aeromagnetic data (TAD, AS maps) provided a crucial subsurface structural blueprint, revealing the fault networks that compartmentalize the aquifer into horsts and grabens. DC resistivity survey then validated this framework hydrogeologically, quantifying aquifer thickness, delineating freshwater zones, and pinpointing localized recharge points associated with fault conduits.

Therefore, this multidisciplinary methodology is not merely an enhancement but a necessity for de-risking groundwater exploration and enabling sustainable resource development in arid, basement-influenced terrains. It provides a robust framework for bridging the scale gap between regional reconnaissance and local precision drilling. Ultimately, effective water resource management strategies in such environments must be founded on a holistic understanding of the structural geology, as it is the primary architect of the groundwater system.

The global applicability of this integrative framework extends well beyond the Wadi Hodein basin. In arid and semi-arid regions worldwide (e.g., the basement aquifers of sub-Saharan Africa and the Arabian Shield to the crystalline terrains of India and South America), similar challenges persist: groundwater occurrence is controlled by subsurface structures that remain undetected by surface observations alone. The current exploration framework, combining open-source remote sensing data (e.g., S1A, ALOS-DEM, IMERG) with targeted geophysical surveys (aeromagnetic and resistivity), offers a cost-effective and transferable framework for groundwater exploration in data-sparse regions. By systematically moving from regional-scale probabilistic mapping to local-scale structural validation, this approach can be adapted to diverse geological settings, providing water resource managers with a robust tool for sustainable development in some of the world’s most water-stressed environments.

To further enhance the accuracy and applicability of this framework, several avenues of future research are recommended to address the inherent uncertainties identified in this study. First, the acquisition of high-resolution remote sensing data and land-based geophysical surveys (land magnetic/FDEM sensors) is recommended to refine the mapping of shallow subsurface structures and reduce resolution-related uncertainties. Additionally, comprehensive hydrogeochemical and isotopic fingerprinting (stable isotopes δ^18^O, δ^2^H; age tracers ^14^C, ^3^H) across all identified compartments is essential to quantify recharge sources, confirm hydraulic connectivity, and validate the conceptual flow model. Furthermore, the development of transient numerical groundwater flow models (e.g., MODFLOG, FEFLOW) calibrated with time-series water level data would enable quantification of recharge rates, storage volumes, and sustainable yields under different development scenarios. Finally, targeted exploratory drilling with full-borehole geophysical logging is recommended to ground-truth surface interpretations and provide precise hydrogeological parameters for model calibration. Implementing these recommendations will transform the current static structural model into a dynamic, quantitative tool for long-term water resource management in structurally controlled arid region aquifers.

## Supplementary Information

Supplementary Information.

## Data Availability

All geophysical and remote sensing data used in this study, along with the conditioning layers (or a sufficient subset), class breaks, weight tables, lineament-extraction parameters, model outputs, and main processing parameters, are openly available in the Zenodo repository under 10.5281/zenodo.21502023.
