# Supplementary material for: Enhancing groundwater potential mapping in arid regions through integrated microwave remote sensing, statistical analysis, and geophysical-based modeling
Source: Sci Rep. 2026 Aug 16;16:25527. doi: 10.1038/s41598-026-64619-0 (PMC13478504; doi:10.1038/s41598-026-64619-0)
Supplement: Supplementary file 1 [file 41598_2026_64619_MOESM1_ESM.docx]

**Supplementary Materials**

**Table S1. Pearson correlation coefficients (r) among conditioning factors based on WoE contrast values.**

| Factor | Slope | TWI | Curvature | DTS | SD | Lithology | Rb | LD | LSID | Rainfall |
| --- | --- | --- | --- | --- | --- | --- | --- | --- | --- | --- |
| Slope | 1.00 |  |  |  |  |  |  |  |  |  |
| TWI | **-0.82*** | 1.00 |  |  |  |  |  |  |  |  |
| Curvature | 0.23 | -0.19 | 1.00 |  |  |  |  |  |  |  |
| DTS | **0.67** | **-0.63** | 0.08 | 1.00 |  |  |  |  |  |  |
| SD | **0.58** | **-0.54** | 0.11 | **0.71** | 1.00 |  |  |  |  |  |
| Lithology | 0.41 | -0.38 | 0.06 | 0.44 | 0.47 | 1.00 |  |  |  |  |
| Rb | **0.73** | **-0.68** | 0.15 | **0.59** | **0.52** | 0.49 | 1.00 |  |  |  |
| LD | 0.35 | -0.32 | 0.04 | 0.38 | 0.41 | 0.43 | 0.39 | 1.00 |  |  |
| LSID | 0.33 | -0.30 | 0.03 | 0.42 | 0.45 | 0.40 | 0.38 | **0.86** | 1.00 |  |
| Rainfall | 0.28 | -0.26 | 0.05 | 0.31 | 0.33 | 0.24 | 0.29 | 0.34 | 0.32 | 1.00 |

**Values in* ***bold*** *indicate strong correlation (|r| > 0.7) [81]*

**Table S2. Variance Inflation Factor (VIF) for each conditioning factor.**

| **Factor** | **R²** | **VIF** | **Interpretation** |
| --- | --- | --- | --- |
| **Slope** | 0.78 | 4.55 | Moderate (acceptable) |
| **TWI** | 0.74 | 3.85 | Moderate (acceptable) |
| **Curvature** | 0.08 | 1.09 | Low (independent) |
| **DTS** | 0.69 | 3.23 | Moderate (acceptable) |
| **SD** | 0.66 | 2.94 | Moderate (acceptable) |
| **Lithology** | 0.51 | 2.04 | Low |
| **Rb** | 0.71 | 3.45 | Moderate (acceptable) |
| **LD** | 0.77 | 4.35 | Moderate (acceptable) |
| **LSID** | 0.81 | **5.26** | **Moderate-High (exceeds threshold)** |
| **Rainfall** | 0.18 | 1.22 | Low (independent) |

*VIF > 5 indicates moderate-to-severe multicollinearity; VIF > 10 indicates severe multicollinearity requiring correction [83]

Table 3S: Comparison between the aquifer parameters from resistivity soundings (VES), GWp values from WOF, EBF models, and the available borehole information.

| **VES** | **Locations** | | **Aquifer parameters from VES** | | | | | groundwater potentiality (GwP)**.** | | | **Borehole information** | | |
| --- | --- | --- | --- | --- | --- | --- | --- | --- | --- | --- | --- | --- | --- |
|  | East | North | Resistivity (Ω.m) | Thickness (m) | Depth (m) | Lithology | WOE- | | EBF | Well depth (m) | | TDS (ppm) |  |
| **1** | 34' 42' 42.' | 23' 18' 44.' | 177/550 | 14.2 | 3 | Nubian Sandstone / fractured basement | High | | High |  | |  |  |
| **2** | 34' 42' 24' | 23' 18' 53' | 46 | 22 | 17.8 | Nubian sandstone | High | | High |  | |  |  |
| **3** | 34' 42' 18' | 23' 19' 50' | 78.8 | 75 | 31.5 | Nubian sandstone | V. High | | V. High |  | |  |  |
| **4** | 34' 42' 9' | 23' 19' 1' | 111 | 65 | 30 | Nubian sandstone | V. High | | V. High |  | |  |  |
| **5** | 34' 42' 14' | 23' 18' 32' | 119/371 | 85 | 7.8 | Nubian Sandstone / fractured basement | V. High | | V. High | 50 | |  |  |
| **6** | **34°48'32** | 23°17' 57 | 120 | 30 | 30.4 | Nubian sandstone | V. High | | V. High | 90.53 | | 824 |  |
| **7** | 34' 42' 34' | 23' 17' 44' | 151 | 10 | 2.3 | Nubian sandstone | High | | High |  | |  |  |
| **8** | 34' 42' 55' | 23' 17' 53' | 165 | 65 | 58.5 | Nubian sandstone | V. High | | V. High | 155 | | 915 |  |
| **9** | **34°44'55** | 23°18'47 | 560 | 21 | 5.6 | Fractured basement | High | | High |  | |  |  |
| **10** | 34' 46' 56' | 23' 17' 48' | 83.5 | 10.8 | 2.9 | Nubian sandstone | High | | High | Spring | | 800 |  |
| **11** | 34' 49' 15' | 23' 18' 34' | 17.5 | 29.7 | 31.6 | Nubian sandstone | V. High | | High | 83 | | 2120 |  |
| **12** | 34' 55' 43 | 23' 15' 8' | 227 | 110 | 31.5 | Nubian sandstone | V. High | | High | 134 | | 568 |  |
| **13** | 34' 56' 6' | 23' 15' 50' | 76.8 | 65 | 48.5 | Nubian sandstone | High | | High | 90 | | 1061 |  |
| **14** | 34' 53' 36.' | 23' 18' 40' | 71.4 | 21 | 12.5 | Fractured Metamorphic | Medium | | Medium |  | | 7931 |  |
| **15** | **34°51'37** | 23°22'36 | 275 | 10.2 | 2.3 | Fractured Granite | Medium | | Low |  | | 713 |  |
| **16** | **34°55'03** | 23°22'42 | 556 | 10.2 | 1.5 | Fractured basement | low | | low |  | | 2237 |  |
| **17** | 34' 52' 18.' | 23' 25' 23' | 246 | 28 | 3.3 | Nubian sandstone | High | | High | Spring | | 412 |  |

**Fig. S1:** Representative resistivity curves with their interpretations and corresponding RMS% errors. The curves display substantial variability in curve shapes, resistivity values, and layer thicknesses, consistent with the local geological complexity influenced by subsurface faults and basement relief. For the locations of the corresponding VESs, see **Fig. 5**.
